# Early Host Responses of Seasonal and Pandemic Influenza A Viruses in Primary Well-Differentiated Human Lung Epithelial Cells

**DOI:** 10.1371/journal.pone.0078912

**Published:** 2013-11-14

**Authors:** Rachael L. Gerlach, Jeremy V. Camp, Yong-Kyu Chu, Colleen B. Jonsson

**Affiliations:** 1 Department of Microbiology and Immunology, University of Louisville, Louisville, Kentucky, United States of America; 2 Center for Predictive Medicine for Biodefense and Emerging Infectious Diseases, University of Louisville, Louisville, Kentucky, United States of America; Johns Hopkins University – Bloomberg School of Public Health, United States of America

## Abstract

Replication, cell tropism and the magnitude of the host's antiviral immune response each contribute to the resulting pathogenicity of influenza A viruses (IAV) in humans. In contrast to seasonal IAV in human cases, the 2009 H1N1 pandemic IAV (H1N1pdm) shows a greater tropism for infection of the lung similar to H5N1. We hypothesized that host responses during infection of well-differentiated, primary human bronchial epithelial cells (wd-NHBE) may differ between seasonal (H1N1 A/BN/59/07) and H1N1pdm isolates from a fatal (A/KY/180/10) and nonfatal (A/KY/136/09) case. For each virus, the level of infectious virus and host response to infection (gene expression and apical/basal cytokine/chemokine profiles) were measured in wd-NHBE at 8, 24, 36, 48 and 72 hours post-infection (hpi). At 24 and 36 hpi, KY/180 showed a significant, ten-fold higher titer as compared to the other two isolates. Apical cytokine/chemokine levels of IL-6, IL-8 and GRO were similar in wd-NHBE cells infected by each of these viruses. At 24 and 36 hpi, NHBE cells had greater levels of pro-inflammatory cytokines including IFN-α, CCL2, TNF-α, and CCL5, when infected by pandemic viruses *a*s compared with seasonal. Polarization of IL-6 in wd-NHBE cells was greatest at 36 hpi for all isolates. Differential polarized secretion was suggested for CCL5 across isolates. Despite differences in viral titer across isolates, no significant differences were observed in KY/180 and KY/136 gene expression intensity profiles. Microarray profiles of wd-NHBE cells diverged at 36 hpi with 1647 genes commonly shared by wd-NHBE cells infected by pandemic, but not seasonal isolates. Significant differences were observed in cytokine signaling, apoptosis, and cytoskeletal arrangement pathways. Our studies revealed differences in temporal dynamics and basal levels of cytokine/chemokine responses of wd-NHBE cells infected with each isolate; however, wd-NHBE cell gene intensity profiles were not significantly different between the two pandemic isolates suggesting post-transcriptional or later differences in viral-host interactions.

## Introduction

The 2009 pandemic H1N1 influenza virus (H1N1pdm) arose through reassortment of two preexisting swine influenza viruses, a Eurasian avian-like virus and a North American triple reassortant virus [1], [2]. Epidemiological data illustrated the speed of global spread of the 2009 pandemic virus; including significantly high infection attack rates in children and an 80% of H1N1pdm deaths in people younger than 65 years of age [3]. This was unlike seasonal influenza A virus (IAV) where morbidity and mortality are mainly seen in the elderly [4]. The illness associated with H1N1pdm infection was, however, very similar to seasonal influenza [5]. The risk factors associated with human cases of H1N1pdm mirrored those of seasonal influenza [3], although in contrast to seasonal influenza, a greater proportion of severe and fatal cases had a pre-existing chronic illness [3], [4], [6]. The most common underlying chronic conditions among hospitalized patients were respiratory disease, asthma, cardiac disease, and diabetes [3], [5], [7]. Immunohistopathology of patients with lethal disease confirmed positive for H1N1pdm identified the major cellular targets of infection as being upper respiratory epithelial cells, type II pneumocytes, and occasionally macrophages, which is similar to the pattern previously observed in H5N1 cases [8].

Most seasonal IAV strains infect primarily the upper respiratory tract with limited lower respiratory tract involvement. The ability of H1N1pdm viruses to infect the lungs within lower respiratory track has been attributed to a broader specificity in the binding of the H1N1pdm surface hemagglutinin (HA) with the α2→3-linked sialic acid (SA) (common on ciliated cells) and α2→6-linked SA (common on non-ciliated secretory cells) [9]–[12]. There are mixed conclusions in the field regarding what cell type is “readily” infected by seasonal influenza strains, with data supporting both ciliated and non-ciliated cell infections [10], [13]. Seasonal IAV and H1N1pdm viruses enter and replicate efficiently into nonciliated cells which are present in the epithelial cell layer in both the large and small airways of the lower respiratory tract, while H5N1 enters and replicates more efficiently in ciliated cells within the small airways [9], [11], [14]–[17]. Hence, the spatial distribution and concentration of potential receptors associated within different areas of the respiratory tract and/or different cell types are integral in the study of IAV infection and disease [9], [18]–[21]. Further, while the lung epithelium is a primary target for infection [22], it is a highly complex environment composed of a heterogeneous cell population, including secretory (Clara), goblet (mucus), ciliated, and basal cells that differ in frequency and distribution depending on location in the lung [23]. The use of polarized, primary cell culture models that contains both types of sialic acid receptors and represent a more comprehensive model for infection are important to the advancement of our understanding of virus-host interactions such as those that modulate the outcome of IAV infection and disease [10], [15], [17], [24], [25].

Early host responses elicited by IAV of host epithelial cells likely control the magnitude, duration and lethality of infection. Once infected by IAV, cells respond by eliciting antiviral response genes and pro-inflammatory/chemotactic cytokines and chemokines [26], [27]. This initial innate immune response is triggered by pattern recognition receptors (PRRs) within the cell. PRR pathways further activate intracellular signaling cascades, such as nuclear factor-kappa beta (NF-κB) and mitogen-activate protein kinase (MAPK). Activation of these pathways leads to the induction of inflammatory cytokines and type I interferon (IFN) secretion. This further stimulates the antiviral signals through IFN-stimulated genes (ISGs) [28]–[33]. Pro-inflammatory cytokine and chemokine products are critical responses as they are important for recruiting immune cells to the site of infection that are key to clearing the virus, as well as activating the adaptive immune response [34], [35]. Of importance, infection of human epithelial cells with H1N1pdm virus have shown a diminished induction of innate immune responses as compared to seasonal H1N1 [20]. Notably, recent findings suggest isolate-specific differences among H1N1pdm viruses as shown by their ability to induce varying degrees of early host antiviral and inflammatory responses in human respiratory epithelial cells [21].

To probe potential differences in early infectivity and host responses of cells infected with seasonal or pandemic IAVs, we utilized a polarized, model of primary, well-differentiated normal human bronchial epithelial (wd-NHBE) cells. We hypothesized that early stages of infection in the airway epithelium may differ in terms of replication and host immune responses between a H1N1 seasonal isolate (A/BN/59/07) and two H1N1pdm strains shown to have fatal (A/KY/180/10) and nonfatal (A/KY/136/09, A/BN/59/2007) outcomes in hospitalized patients (Table 1) [36]. The two H1N1pdm clinical isolates (KY/180 and KY/136) differ in their pathogenicity and cytokine/chemokine profiles in a DBA/2 mouse model [36]. In this study, we demonstrate a comparison of infection of wd-NHBE cells with each IAV isolates show differences in virus titers and the dynamics of the host cytokine and chemokine responses. We show that infection with the lethal H1N1pdm isolate (KY/180) alters the structure and cellular integrity of the epithelial layer, replicates more efficiently, and results in an increased, polarized pro-inflammatory cytokine and chemokine responses. Interestingly, the microarray profiles of the antiviral signaling pathways do not correlate with differences in the virus titer of host cytokine and chemokine responses. This suggests that post-transcriptional events may mediate the isolate-specific nature of the host cytokine and chemokine responses.

**Table 1:** Seasonal and pandemic IAV isolates used in this study.

Virus	Subtype	Source	Phenotype
KY/180	H1N1pdm	Fatal Case	Lethal
KY/136	H1N1pdm	Non-fatal Case	Non-lethal
BN/59	Seasonal H1N1	CDC	Non-lethal

## Methods

### Viruses and cells

The 2009 H1N1pdm IAV strains used herein were A/Kentucky/180/2010, (KY/180), and A/Kentucky/136/2009, (KY/136), from nasal swabs taken from a fatal and non-fatal case, respectively [36]. The GenBank accession numbers for KY/180 and KY/136 are provided in Table S1. The seasonal H1N1 IAV vaccine strain A/Brisbane/59/2007 (BN/59) was kindly provided by the Centers for Disease Control and Prevention, Virus Surveillance and Diagnosis Branch, Influenza Division. Viral seed stocks were prepared as previously described [36]. Virus titers were determined by TCID_50_ (50% tissue culture infectious dose) using MDCK (Madin-Darby Canine Kidney Epithelial Cells) as described previously [36] and calculated using the method of Reed and Muench [37].

All cell culture reagents were purchased from Invitrogen unless otherwise noted. The human lung bronchial epithelial (Calu-3), human adenocarcinomic alveolar basal epithelial (A549), and MDCK epithelial cells (ATCC) were cultured in Dulbecco's minimum essential medium supplemented with 5 mM L- glutamine, 1% pen-strep and 10% fetal bovine serum at 37°C under 5% CO_2_. Undifferentiated (ud-NHBE) cells were purchased from Lonza and cultured according to the suppliers instructions in serum-free, hormone supplemented bronchial epithelial growth media.

Primary wd-NHBE cells (EpiAirway PC-12, MatTek Corporation) were shipped in 12-well plates with agarose embedded in the basal layer and air apically after being maintained for 28 days under an air-liquid interface. Upon arrival, the transwell inserts were removed and placed into a 12-well plate with media in the basal compartment (AIR 100 complete growth media, MatTek). No media was added to the apical layer. Cells were incubated at 37°C, 5% CO_2_ and the basal media was changed after 24 h. At this point the cells were ready for infection and this is described in the next section.

### *In vitro* IAV infection

Infection of continuous and primary cells lines were performed in triplicate for measurement of virus production, immune responses or microarray studies. Each experiment (except for microarray) was replicated three times. Calu-3, A549, MDCK and ud-NHBE cells were infected with KY/180, KY/136, BN/59 or mock-infected (using viral growth media as specified in prior section) at a multiplicity of infection (MOI) of 3 for 1 h at 37°C, 5% CO_2_. IAV infection of Calu-3, A549 or MDCK included 2 µg/ml of tosylsulfonyl phenylalanylchloromethyl ketone-treated trypsin (Sigma) and 0.2% BSA in the media.

Wd-NHBE cells were washed twice with Dubelcco's phosphate buffered saline (DPBS) to remove mucus accumulation and infected at an MOI of 3 in triplicate in replicate experiments from a total of three donors. After 1 h, the apical layer was washed twice with DPBS to remove unbound virus. Basal medium was removed and replaced with complete medium. At each time point analyzed, the basal media was removed and apical layer washed twice with 0.5 ml DPBS supplemented with 0.2% BSA and stored at −80°C until use. Cells were collected in TRIzol and stored at −80°C until used for RNA and protein extraction.

### Quantitative RT-PCR (qRT-PCR)

Total RNA from each set of viral-infected cells was extracted at designated time points using TRIzol as described by Invitrogen. cDNA was synthesized from total RNA with random hexamer primers and Superscript III reverse transcriptase (Invitrogen). Gene specific primers were used to amplify the HA genomic RNA using SYBR green select (Invitrogen) and detected with a 7900HT Real-time PCR System (Applied Biosystems). The amount of HA copy number was determined by extrapolating the Ct of each replicate against the standard curve generated using 10-fold dilutions of HA plasmid with known copy number. The sequences of the forward primers for H1N1pdm were 5′-CACCAGTCCACGATTGCAATA-3′ and for BN/59 5′-GAGTAGAGGCTTTGGATCAGGA-3′. The reverse primer was the same for both H1N1pdm and seasonal (5′-ATGGGAGGCTGGTGTTTATAGC-3′).

### Quantification of apical and basal levels of virus and immune responses in wd-NHBE cells

Virus titers and cytokine/chemokine protein levels were measured in basal and apical supernatants in two experiments with two donors. Virus titer was measured by TCID_50_ as discussed above. We measured levels of CCL2/MCP-1, CCL5/RANTES, IL-6, CXCL8/IL-8, G-CSF, GM-CSF, CXCL1/GRO, IFN-α, CCL4/MIP-1β, CXCL10/IP-10, IL-10, and TNF-α using multiplexed arrays according to the manufacturer's protocol (Millipore) using a Luminex 100™ machine. Concentrations for each secreted cytokine and chemokine were determined using standard curves and Luminex xPONENT® software.

### Microarray studies

Total RNA was extracted by TRIzol from three replicates of virus-infected or mock-infected wd-NHBE cells from a single donor and further purified using RNeasy kit (Qiagen). The samples were run in triplicate on Affymetrix HG-U133 plus 2.0 chips (Affymetrix) and processed according to the manufacturer in the Microarray Core facility at the University of Louisville. The raw data have been deposited in a Gene Express Omnibus (GEO). The GEO accession number is GSE48466. Prior to statistical analyses, raw data were processed by Plier Workflow normalization method using Gene Console software (Affymetrix, version 1.3.1). After normalization, data were log_2_ transformed and differentially expressed genes (DEGs) were identified by one-way analysis of variance (ANOVA) using Partek Genomics Suite 6.5 software. Fold-change and p-values were calculated for each virus infection, as compared to the mock-infected. Principal component analysis was conducted as a quality control measure to ensure the three replicates per viral treatment grouped together with limited variation (Figure S1). The data set was further filtered to select statistically significant genes and corrected using a p-value of 0.05 with a 2-fold cut-off. Data filtering and pathway analyses were performed using Ingenuity Pathway Analysis (IPA, Ingenuity Systems) software.

### Immunohistochemistry

Tissues were fixed in 10% buffered formalin, processed, and paraffin embedded. Four-micron thick sections from infected wd-NHBE cells at 36 hpi were processed by immunohistochemistry (IHC). Antigen retrieval and staining of the paraffin-embedded sections of the wd-NHBE cells were performed as others described [38]. Briefly, paraffin was removed; sections were incubated with pronase and blocked with H_2_O_2_ in Tris-buffered saline and with avidin/biotin blocking kit (Vector Labs). The slides were incubated with primary antibody specific for NP protein (East Coast Bio), blocked with goat serum, and then incubated with VECTASTAIN ABC kit (Vector Labs). Development was then performed using either diaminobenzidine (Vector Labs), and secretory cells were counterstained with Alcian Blue (Sigma) and mounted with Permount (Fisher). Cell layers were measured using Zeiss software measurement tool using a 10X objective. Five pictures with 3–4 measurements per picture were taken. Five images per slide were used to quantitate cell layer thickness (3–4 segments/image).

### Statistical analysis

The differences of log_10_-transformed viral titers among different viruses at different time points post-infection and the quantitative cytokine and chemokine mRNAs and proteins of influenza virus-infected cells were compared by using one-way ANOVA followed by a Bonferroni multiple-comparison test, unless otherwise stated. Differences were considered statistically significant at a *p*-value less than or equal to 0.05. The statistical analysis was performed using Graph-Pad Prism 5.04 and Partek Genomics Suite 6.5 software.

## Results

### Kinetics of viral replication of pandemic and seasonal H1N1 isolates in continuous and primary cell lines

To select a cell type for microarray and cytokine studies, we used several cell types (primary and continuous) to screen for potential differences in the ability to infect and produce infectious virus among the pandemic (KY/180 and KY/136) and seasonal (BN/59) isolates. We chose Calu-3, A549, ud-NHBE, and wd-NHBE cells and an MOI of 3 for this study. We included primary cell lines (ud-NHBE and wd-NHBE cells) to ascertain if a more complex cell culture model would reveal greater differences. Differences in entry were anticipated as the KY/180 has a D222G signature in the HA [36]. The ud-NHBE was included to determine the general influence of the α2→3-linked SA (common on ciliated cells) in the wd-NHBE cells as compared to the α2→6-linked SA (common on non-ciliated secretory cells) in the ud-NHBE cells. Supernate was collected over 3 days to measure the kinetics of each virus with the TCID_50_ assay.

**Figure 1:**
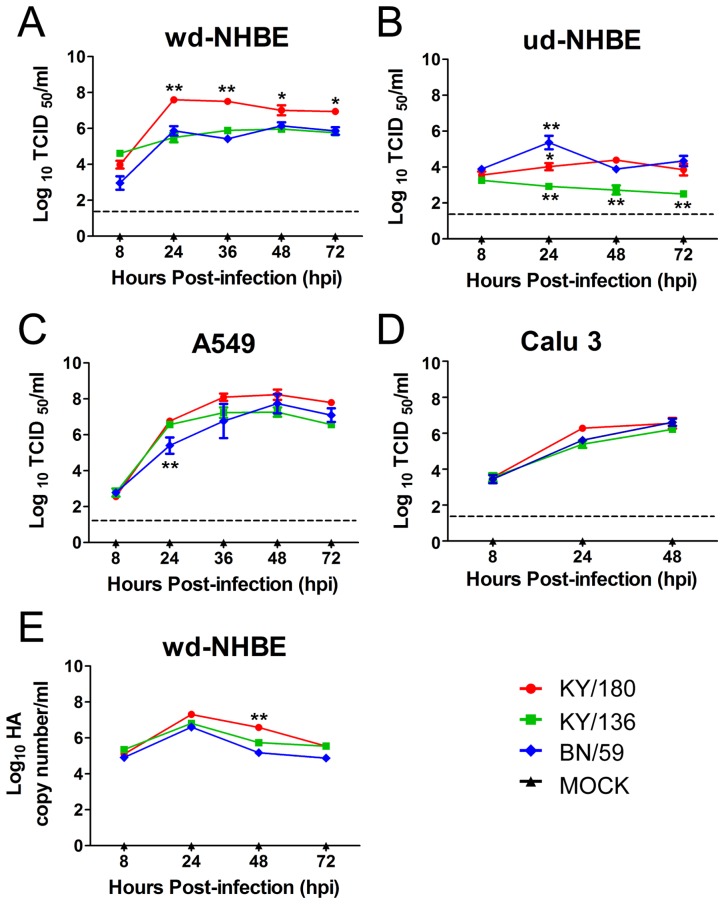
Virus titer detected in supernatant from cells infected seasonal and pandemic IAVs. (A) wd-NHBE, (B) ud-NHBE, (C) A549, and (D) Calu-3 cells were infected with 3 MOI of seasonal (BN/59) or pandemic (KY/180, KY/136) viral isolates and apical wash from wd-NHBE cells and supernatants from ud-NHBE, A549, and Calu3 cells were collected at 8, 24, 36, 48, and/or 72 hpi. The virus titer was determined using a TCID_50_ assay. In (E), the amount of viral HA RNA in cells was quantified by qRT-PCR wd-NHBE cells using the Ct method. Data are presented as the mean ± SEM of the virus titer pooled from 3 replicates from three independent experiments with 3 donors (A–D) or 1 donor (E). Asterisks indicate significance of p<0.05 (*), p<0.01 (**), and p<0.001 (***) respectively. The dotted line indicates the limit of detection of the TCID_50_ assay.

All three isolates infected and produced infectious virus in the all cell types apically (Figure 1A–D). No virus was detected in basal supernatant of infected cells across all time points (data not shown). The wd-NHBE as compared to the ud-NHBE cells conferred a distinct advantage showing a 2–3 fold higher level production of infection virus over time suggesting the importance of the α2→6-linked SA (Figure 1A versus 1B). In the primary wd-NHBE cells, the titer of all three isolates peaked at 24 hours post-infection (hpi) (Figure 1A). KY/180 showed significantly higher levels of virus at 24 hpi than KY/136 and BN/59 apically (Figure 1A). In ud-NHBE cells, significant differences occurred between isolates over time (Figure 1B). In the A549 cells, viruses peaked at 36–48 hpi at the highest levels of any of the cells (Figure 1C). Pandemic isolates replicated more efficiently at 24 hpi as compared to the seasonal isolate (Figure 1C). In the Calu-3 cells, no significant differences in replication occurred between isolates over the time course of infection (Figure 1D). Given the greater differences between KY/180 and the other viruses, the primary wd-NHBE cell, a physiologically relevant model, was chosen for further analyses. The level of viral RNA as measured by the HA was assessed in NHBE to further explore the difference in viral titer. The viral RNA levels were similar among all three isolates at 24 hpi suggesting that another mechanism was responsible for the higher levels of virus such assembly of budding.

**Figure 2:**
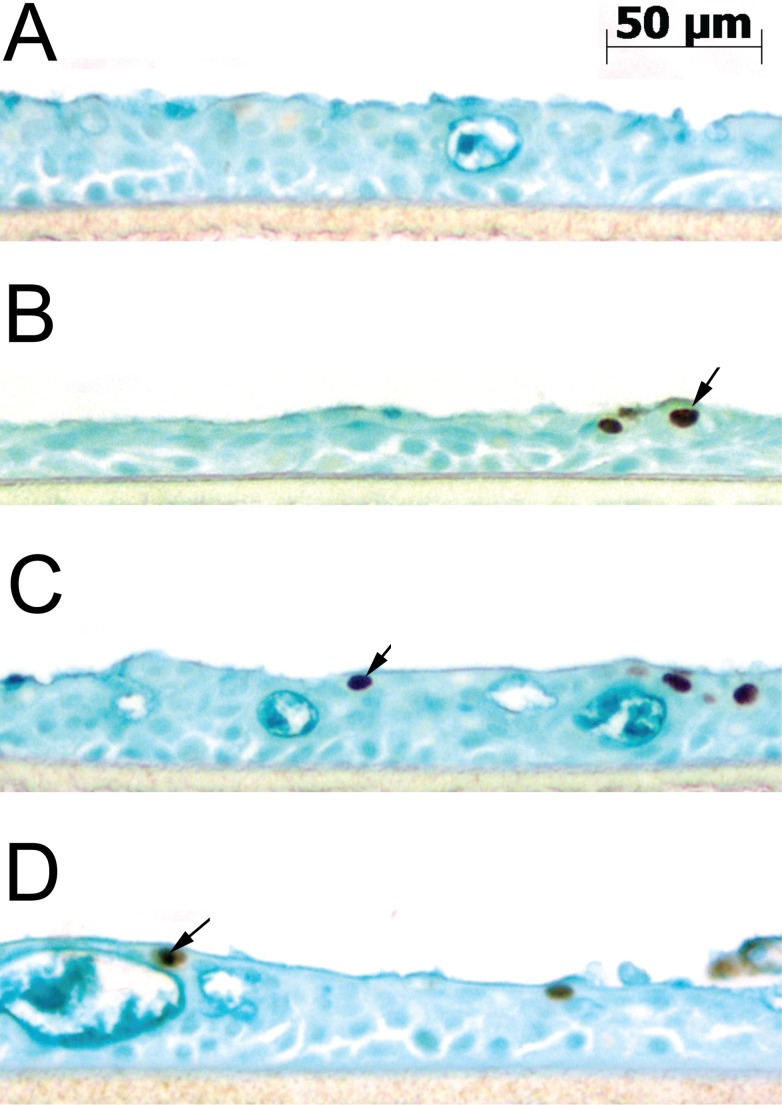
Immunohistochemical microscopy of wd-NHBE cells after IAV infection. IHC microscopy of wd-NHBE cells stained with Alcian blue and evaluated 36 h after infection with (A) MOCK, (B) KY/180, (C) KY/136, and (D) BN/59 for localization of influenza nucleoprotein (NP) antigen (brown) in the epithelial cell nucleus.

Infection of the wd-NHBE cells was confirmed by IHC for each isolate as compared to mock-infected at 36 hpi (Figure 2). The 36 hpi time point was chosen based on preliminary studies measuring the level of IFN-β which peaked at 36 hpi (data not shown) coupled with the differences in the viral titer data. Staining for IAV nucleoprotein (NP) showed a similar distribution of infected cells for all three isolates.

### Cytokines and chemokines elicited in wd-NHBE cells by H1N1 IAV isolates show different trajectories

**Figure 3:**
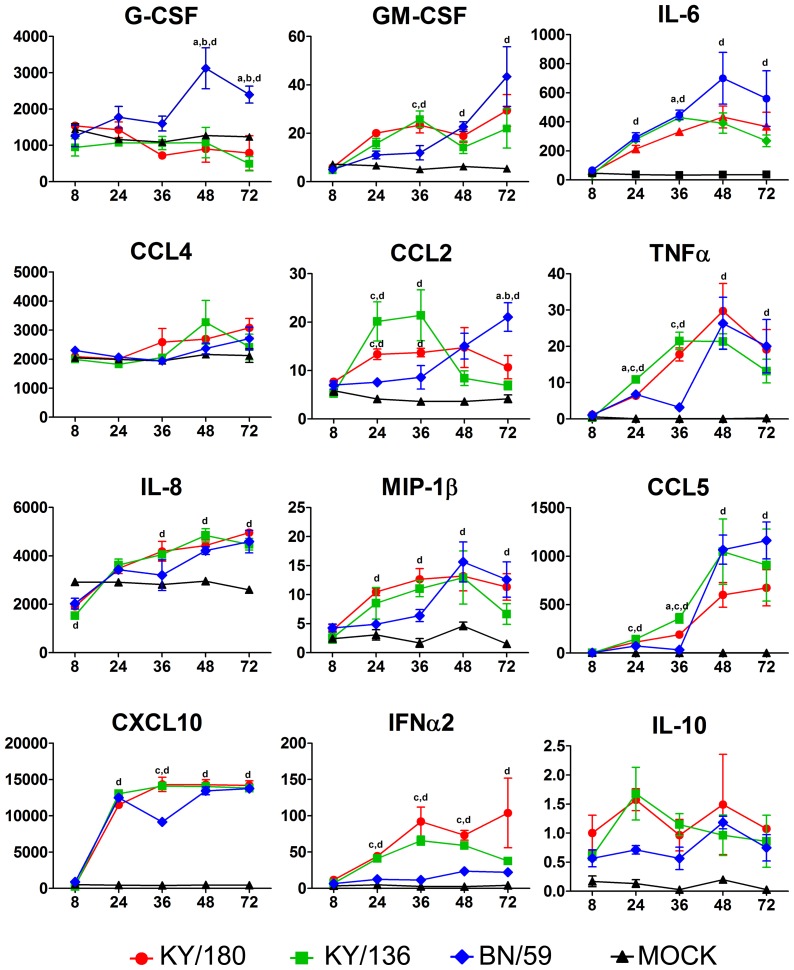
Apical cytokine and chemokine production by wd-NHBE cells infected with seasonal and pandemic IAVs. After infection, the apical side of the culture insert was washed twice and harvested for Luminex multiplex analysis. The error bars indicate mean ± SEM from 3 replicates per isolate per time point from one representative experiment. A total of two experiments were conducted with two donors. Letters indicate significant differences between isolates (a- different from KY/180, b- different from KY/136, c- different from BN/59, and d- different from Mock).

**Figure 4:**
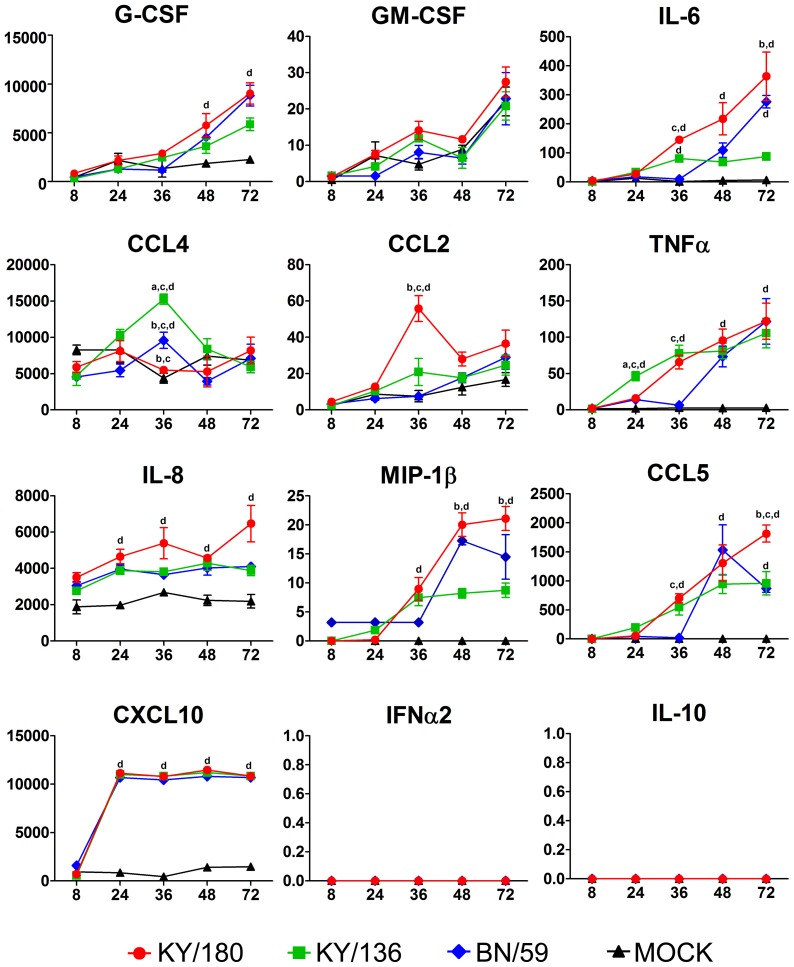
Basal cytokine and chemokine production by wd-NHBE cells infected with seasonal and pandemic IAVs. After infection cell culture supernatants were harvested from the basal side of the culture insert and a multiplex analysis was performed using Luminex platform. A total of two experiments were conducted with two donors. The error bars indicate mean ± SEM from 3 replicates per isolate per time point from one representative experiment. Letters indicate significant differences between isolates (a- different from KY/180, b- different from KY/136, c- different from BN/59, and d- different from Mock).

**Figure 5:**
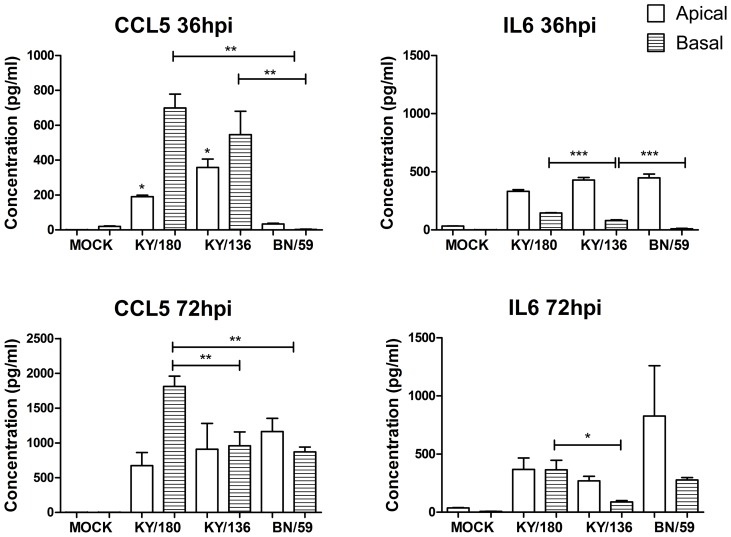
Apical and basal secretion of cytokines and chemokines in wd-NHBE cultures infected with seasonal and pandemic IAV at 36 hpi. Culture supernatants were harvested from the apical and basal side of the culture inserts and screened for presence of protein using Luminex platform. The error bars indicate mean ± SEM from 3 replicates per isolate per time point from one representative experiment. The mean and SEM from 3 replicates per isolate per time point are shown. Asterisks indicate significance of p<0.05 (*), p<0.01 (**), and p<0.001 (***).

Differences in replication among the three H1N1 isolates prompted us to ask whether differences occurred in the levels of cytokine and chemokine secreted from the infected wd-NHBE cells. We analyzed the levels of 12 cytokines and chemokines over time in the apical and basal medium (Figure 3 and 4). At 24 and 36 hpi, both H1N1pdm isolates showed greater levels of pro-inflammatory markers, apically (CCL5, GM-CSF, CXCL10, MCP-1, CCL4) and basally (CCL5, IL-6, TNF-α), compared to BN/59 (Figure 3 and 4). The concentration of apical IL-6, IL-8 and GRO secreted by cells were similar between all isolates (Figure 3). IFN-α was secreted apically, and not basally, in cells infected by pandemic or seasonal isolates. IL-10 occurred in trace amounts apically and was absent basally in all three isolate infected cultures. Overall, the patterns were fairly similar for pandemic isolates in the apical wash. Significant differences were seen between isolates in the basal culture supernatants (Table S2–4). The only notable differences between KY/180 and KY/136 were the greater levels of CCL2, Il-8, IL-6 and CCL5 in the basal media at 36 and 72 hpi (Figure 5).

### Microarray analyses of NHBE cells infected with seasonal or pandemic isolates

**Figure 6:**
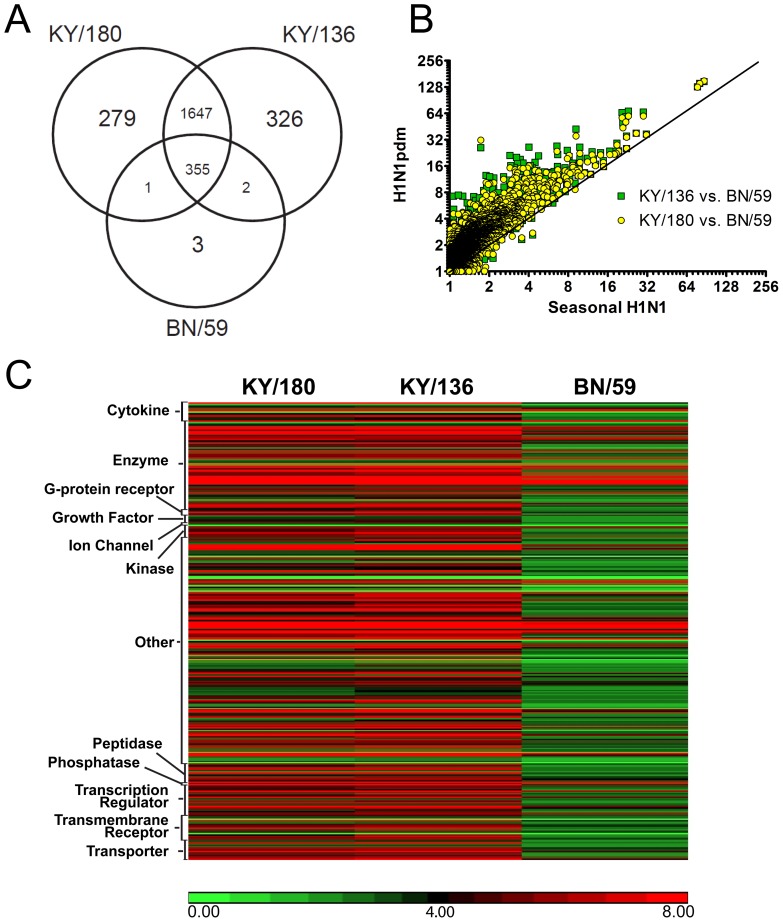
Microarray analysis. Differentially expressed genes (DEGs) were identified by one-way ANOVA analysis by comparing mock and IAV-induced gene expression intensities in wd-NHBE cells. DEGs were selected by filtering the genes whose expression changed by at least 2-fold relative to the level in the mock infected group with a p<0.05, as outlined in *Materials and Methods*. (A) The Venn diagram illustrates the agreement between the lists of DEGs detected by microarray. (B) Overall data are represented in scatter plots of log-2 fold-change expression data of seasonal vs. pandemic infected cells at 36 hpi. The diagonal line indicates where the fold change values would be equivalent for the compared isolates. (C) Gene expression intensities were visualized by means of a heatmap of the 355 differentially expressed genes common to all three isolates. Clusters represent types of genes as defined by the Ingenuity pathway analysis output. The error bars indicate mean ± SEM from 3 replicates per isolate per time point from a single donor. Asterisks indicate significance of p<0.05 (*), p<0.01 (**), and p<0.001 (***) respectively.

To complement our cytokine and chemokine studies, we measured differences in gene transcription levels at 36 hpi by microarray. Overall, cells infected with KY/180 or KY/136 had roughly 2,000 genes that were significantly up- or down-regulated as compared to mock-infected, whereas the seasonal BN/59 isolate had only 360 genes significantly up- or down-regulated (Table 2). A Venn diagram shows the agreement between the three lists of genes (Figure 6A). There were 355 significant DEGs (p<0.05) in wd-NHBE cells common to all three isolates at the 2-fold cut-off (Figure 6A); of which, many of the genes were from the early innate immune response pathways (Table 3). A complete list of genes is provided in Table S5. For all three IAVs the largest category of up-regulated genes was the ISGs (e.g., RSAD2, IFIT2, IFI44L, IFIT3, OAS1, OASL, MX2, STAT1) (Tables 3 and S5). Other genes up-regulated by all three isolated included interferon-induced chemokines (e.g., CCL5, CXCL9, CXCL10, and CXCL11), type III-IFN (e.g., IL29, IL28A and IL1A), PRRs (e.g., DDX58, IFIH1, TLR3, MYD88, CASP1), and other regulatory factors (e.g., IDO1, SOCS, EIF2AK2). Surprisingly, when a 2-fold change cut-off with a significance of p<0.05 was applied there were only three genes unique to BN/59-infected cells (Table S6), whereas KY/180 and KY/136 had 279 and 326 unique DEGs respectively (see the top 25 significant genes in Table S7 and S8).

**Table 2:** Differentially expressed genes in IAV–infected NHBE cells at 36 hpi.

Virus	No. Differentially Regulated Genes*
KY/180	2281
KY/136	2338
BN/59	360

* No. significant genes p<0.05, 2-fold cut-off.

**Table 3:** Notable genes upregulated in wd-NHBE cells infected with seasonal and pandemic IAV isolates at 36 hpi.

Gene Symbol	Affymetrix Probe ID	Fold Change KY/180	Fold Change KY/136	Fold Change BN/59
RSAD2	213797_at	34.23	35.17	22.28
IFIT1	203153_at	25.48	25.28	22.24
IFIT2	226757_at	36.38	37.06	31.47
IFIT3	204747_at	27.81	29.10	18.81
SOCS1	210001_s_at	9.70	13.70	5.30
IFITM2	201315_x_at	8.77	9.13	6.46
IFI35	209417_s_at	16.30	17.24	8.67
IRF1	238725_at	4.18	5.09	3.09
IRF9	203882_at	3.07	3.35	2.64
IFI44L	204439_at	15.81	15.75	14.87
OAS1	205552_s_at	12.21	12.56	7.48
OASL	210797_s_at	57.01	65.33	20.55
MX1	202086_at	17.52	18.42	14.80
MX2	204994_at	22.35	23.55	18.55
JAK2	205842_s_at	4.94	5.65	2.28
STAT1	200887_s_at	4.07	4.16	4.00
STAT2	205170_at	3.14	3.29	2.37
PSMB8	209040_s_at	3.34	3.68	2.76
CCL5	1555759_a_at	15.06	19.06	3.45
CXCL9	203915_at	2.74	2.61	4.29
CXCL10	204533_at	127.36	128.62	77.29
CXCL11	210163_at	140.77	140.60	80.39
IL29	1552917_at	9.91	16.13	2.92
IL28A	1552915_at	12.53	20.10	3.23
IL1A	210118_s_at	4.32	3.98	2.00
DDX58	218943_s_at	21.42	23.25	12.17
IFIH1	219209_at	11.71	12.03	7.72
TLR3	206271_at	5.91	6.32	3.43
CASP1	211367_s_at	4.69	5.16	2.62
MYD88	209124_at	3.54	3.64	2.30
IDO1	210029_at	17.61	19.36	9.83
SOCS2	203373_at	5.73	6.08	2.05
EIF2AK2	204211_x_at	3.55	3.46	3.17

Fold change values obtained by 1-way ANOVA analysis comparing gene expression intensities of seasonal and pandemic IAV-infected cells to mock. Analysis conducted using Ingenuity core analysis (p<0.05, 2-fold change cut-off).

**Figure 7:**
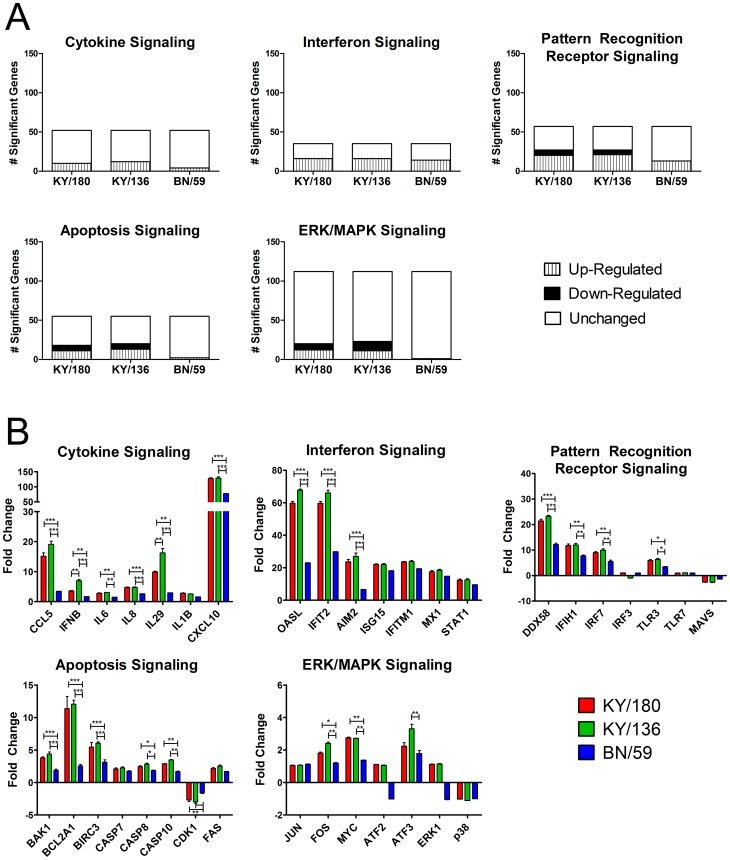
Pathways significantly represented by all isolates as compared to mock. (A) Graphs represent the number of genes differentially up- or down- regulated for each isolate compared to mock. Red represents the number of genes up-regulated, green represents the number of genes down-regulated, and white represents the number of genes that are not significantly different from mock. (B) Graphs represent the fold change expression of significant DEGs within these pathways.

We noted 1647 genes that were commonly expressed in KY/180 or KY/136-infected NHBE cells that were not significant in the BN/59-infected cells (Fig. 6A, Table 2). When comparing global gene expression levels, H1N1pdm-infected wd-NHBE cells showed greater fold-changes in transcription as compared to seasonal IAV (Figure 6B). Cells infected with H1N1pdm isolates had very similar levels of global gene expression with KY/136 showing slightly greater up-regulation at 36 hpi (Figure 6B). Genes common to both KY/180 and KY/136-infected cells but not BN/59 included transcription factors (cMYC, CDK1, SP1, SOX9, and ATF3), keratinocyte factors (KRT24 and KRT6B), defensins (DEFB1), and protein folding proteins (HSPA6). Also significant were genes involved in activating signal transduction pathways through toll like (TICAM) and the chemokine receptors, CCR4 (Table 4) and apoptosis (Table S9). The similarities of KY/180 and KY/136 to each other and their differences to BN/59 are further revealed upon comparison of the raw numbers of genes within the top canonical pathways, IFN signaling and communication, that were up- and down-regulated were similar between KY/136 and KY/180-infected NHBE cells (Figure 7A). Both KY/180 and KY/136 differed with the pattern shown by BN/59-infected NHBE cells (Figure 7A).

**Table 4:** Fold change of significantly differentially expressed genes.

Gene Symbol	Affymetrix Probe ID	KY/180	KY/136	BN/59
KRT24	220267_at	31.65	25.90	1.73
DEFB1	210397_at	8.10	8.41	1.82
KRT6B	213680_at	6.02	5.12	1.43
HSPA6	213418_at	5.82	7.17	1.52
CCR4	208376 _at	3.95	4.00	1.93
BAK1	203728_at	3.80	4.37	1.89
IFNB1	208173_at	3.58	6.90	1.69
TICAM1	213191_at	3.48	3.88	1.77
IL-6	205207_at	2.78	3.06	1.50
MYC	202431_s_at	2.73	2.72	1.38
CDK1	203213_at	−2.67	−3.00	−1.61
ATF3	202672_s_at	2.22	3.30	1.78
GSTA1	203924_at	−12.09	−12.03	−1.21
SOX9	202936_s_at	4.66	5.38	1.97
ICAM1	202638_s_at	2.28	2.27	1.67
SOCS2	200887_s_at	4.07	4.16	4.00

Fold change values obtained by 1-way ANOVA analysis comparing gene expression intensities of IAV-infected cells to mock. Analysis conducted using Ingenuity (p<0.05, 2-fold change cut-off).

We further conducted pathway analyses using the Ingenuity Pathway Analysis (IPA) to identify the intracellular signaling pathways that were most significantly represented in seasonal and pandemic infected cells using Fisher's Exact Test (Table 5). The top two ranking pathways for all three viruses were the same; IFN signaling and communication between innate and adaptive immune cells. The remaining three pathways and ranking differed in importance. The role of PRRs was shared but greatest for KY/136. The importance of the complement system was suggested for only KY/180, while antigen presentation was suggested for KY/136 and BN/59. Finally the aryl hydrocarbon pathway was significant for KY/180 and KY/136 but not BN/59. Combined with the individual gene analysis, the pathway analysis underscores important similarities but resulting gene specific differences.

**Table 5:** Top five significant canonical pathways in IAV-infected NHBE cells at 36 hpi relative to mock.

RANK	A/KY/180/10	A/KY/136/09	A/BN/59/07
1	IFN Signaling Pathway (6.47E-07, 0.471)	IFN Signaling Pathway (3.49E-07, 0.471)	IFN Signaling Pathway (3.36E-15, 0.412)
2	Communication between Immune Cells (3.07E-06, 0.471)	Communication between Immune Cells (4.70E-06, 0.258)	Communication between Immune Cells (6.51E-12, 0.172)
3	Complement System (2.37E-05, 0.424)	Role of PRRs in Recognition of Viruses (1.15E-05, 0.284)	Antigen Presentation Pathway (1.13E-10, 0.275)
4	Role of PRRs in Recognition of Viruses (2.75E-05, 0.284)	Aryl Hydrocarbon Receptor Signaling (7.14E-05, 0.234)	Activation of IRF of Cytosolic PRRs (1.14E-07, 0.175)
5	Aryl Hydrocarbon Receptor Signaling (7.65E-05, 0.241)	Antigen Presentation Pathway (1.04E-04, 0.325)	Role of PRRs in Recognition of Viruses (1.53E-07, 0.137)

Rankings are listed based on statistical significance scored using Fischer's Exact Test (p-value <0.05). For each canonical pathway we report the p-value of Fisher's exact test to measure significance and the proportion of genes in the pathway that were actually significantly represented in the brackets.

### wd-NHBE cell layer integrity changes overtime after infection

**Figure 8:**
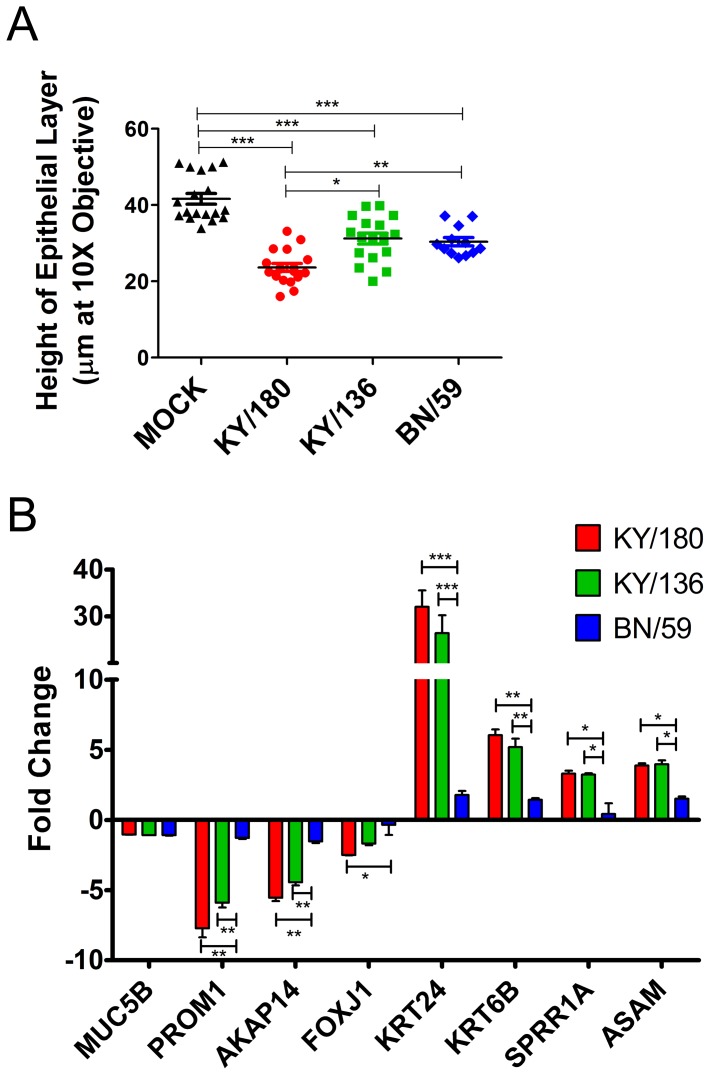
Immunohistochemical microscopy of wd-NHBE cells stained at 36 h after infection the pandemic and seasonal isolates. Cell layers were measured using Zeiss AxioVision version 4.8 software using a 10 X objective. Five pictures were taken with 3-4 measurements per picture. (A) Differences in epithelial layer thickness, as measured by mean height of the epithelial layer from the collagen are depicted. The error bars indicate SEM from 3 replicates per isolate per time point. Asterisks indicate significance of p<0.05 (*), p<0.01 (**), and p<0.001 (***) respectively. (B) Microarray gene expression of genes shown to be associated with differentiation of bronchial epithelial cells and apoptosis. Values are shown as fold-change over mock infected control at the 36 h time point.

When evaluating the cells by IHC, we observed changes in epithelial layer integrity in infected epithelial layers compared to mock-infected wd-NHBE cells (Figure 2). Cultures infected with KY/180 appeared thinner than both mock-infected cells and cells infected with the other IAV isolates (Figure 2). To address this observation, we further analyzed paraffin-embedded sections by measuring the distance from the collagen layer to the top of the epithelial layer (Figure 8A). Significant differences were noted among all isolates compared to the mock-infected control. Cells infected with KY/180 showed the smallest distance, followed by BN/59 and KY/136 as compared to mock (Figure 8A).

Having observed these differences, we turned to the microarray data to determine whether the observed changes in epithelial cell layers could be explained at the transcriptional level (Figure 8B). We evaluated expression levels of DEGs in bronchial epithelial cells after the air-liquid interface culture process. These genes include those associated with cell adhesion, transport, and cilia formation and function, such as SPRR1A, KRT6B, KRT24, ASAM, FOXJ1, MUC5B, AKAP14, and PROM1 (and apoptosis genes CASP7 and BAK1). According to Ross *et al.* (2007) wd-NHBE cells have decreased expression of the keratinocyte marker genes and an increased expression of genes involved in cell signaling, cilia formation and also cilia function [39]. We saw an increase in expression of keratinocyte genes and a decrease in expression of cilia genes in wd-NHBE cells. Cells infected with KY/180 showed a greater difference in gene expression levels over the mock compared to KY/136 and BN/59 (Figure 8B).

## Discussion

The contribution of the early host-virus interactions to the progression of disease remains a critical question. Using *in vitro* models that closely mimic physiological conditions within the lungs in evaluating respiratory infections is an important approach in elucidation of potential differences between strains with different virulence [40], [41]. For example, recent studies evaluating the pathogenesis of 2009 H1N1pdm in bronchial epithelial cells suggest that differentiation status of bronchial epithelial cells has a profound impact on the infection efficiency of different influenza strains and the host innate immune responses [9]. We sought to compare host responses in a wd-NHBE cell culture model to determine whether lung epithelial cells infection differed between seasonal and pandemic influenza isolates.

Recently, Zeng *et al*. evaluated extracellular inflammatory molecules secreted by polarized bronchial epithelial cells (Calu-3) and pharyngeal cells (Detroit 562) infected with 2009 H1N1pdm compared to seasonal. They show the two isolates are considerably different in terms of inflammatory responses, such as type-I IFN, IL-6, CXCL10, and TNF-α, as well as replication efficiency, with H1N1pdm being more efficient [20]. Furthermore, a study comparing different H1N1pdm isolates in ud-NHBE cells show critical differences in levels of cytokines and chemokines elicited from cells infected with closely-related influenza isolates [21]. They show distinct differences in viral infectivity as well as differences in IFN-β levels between 2009 H1N1pdm (CA/08, Mexico/4108, TX/15) and the seasonal H1N1 (Solomon/03) [21]. The differences seen in these models, prompted us to compare the phenotype induced by our genetically, closely-related H1N1pdm and seasonal influenza isolates in wd-NHBE cells.

To select the optimal cell line for microarray and immune response studies to probe regulatory differences among pandemic and seasonal isolated, we screened several continuous and primary cell lines. We show that H1N1pdm isolate KY/180, which was previously reported to be lethal in mice and humans [36], produced significantly more virus in NHBE cells than the other isolates from 24–72 hpi. The ud-NHBE cells were less permissive for production of virus presumably due to less differentiation and lack of the α2→6-linked SA. Previous reports show productive replication of H1N1pdm in NHBE cells [42]. Differences in viral titers among strains of the same HA subtype (i.e., H1) in wd-NHBE cells have not been reported previously. Interestingly, an examination of the viral genomic HA RNA levels did not suggest that this was due to replication levels. Future studies to understand the reason for a higher virus titer will focus on the potential of differences in assembly and/or budding.

Because regulation of innate immunity by viruses is a key determinant of the subsequent host immune response and clinical outcome, we evaluated the cytokine and chemokine secreted apically and basally in wd-NHBE cells. IAV infections lead to a variety of intracellular responses, inducing innate immune signaling cascades which serve as the first line of defense against the invading virus [43]–[45]. Cytokines and chemokines produced by these pathways play an important role in the production of airway inflammation and recruitment of immune cells to the site of infection. A key finding from our data was the greater levels of basolateral secretion of pro-inflammatory cytokines (IL-6, CCL5, IL-8 and CCL2) by wd-NHBE infected with the lethal KY/180 isolates as compared to KY/136 and BN/59. We are aware of only two studies of IAV infection in primary NHBE cells that have looked at secretion of cytokines and chemokines from both the apical and basal side of the epithelial culture [46], [47]. However, these studies were limited to the earliest time points and did not look at the later time points where we saw the greatest differences. We speculate that differences in basolateral signals such as CCL5 from epithelial cells may play a role in the recruitment, activation, and responses elicited by monocytes. Further, the magnitude of the CCL5 response may give rise to differences in outcome [47]. Recently, in mice, apoptosis of virus-infected macrophages was prevented by CCR5/CCL5 [48]. CCL5 has been demonstrated to send an anti-apoptotic signal to the cell via the Akt and Erk1/2 pathways, which could support an increase in survival and scavenging of recruited and resident macrophages.

With replication and apical/basal chemokine/cytokine data suggesting differences among the isolates, we sought to evaluate the intracellular signals gene expression patterns triggered by the virus. These intracellular responses include the double-stranded vRNA recognition by PRRs [49], Nod-like receptors, TLR, and the MAPK pathway, which have all been reported to be important to control of cellular responses against invading pathogens [50]. Three different types of MAPKs, the ERKs, the JNKs, and ERKs, contribute to the generation of cytokines and chemokines, such as IL-8, CCL5, and TNF-α [2]. We hypothesized that differences in up- or down- regulation of genes involved in these pathways would explain the phenotypic differences observed in replication and secretion of cytokines and chemokines in our wd-NHBE infection model. Strikingly, we saw no significant differences in transcriptional profiles between KY/180 and KY/136 within these pathways (Figure S3); indicating a potential for differences in post-transcriptional regulation by KY/180.

IAV have been shown to induce apoptosis *in vitro* and *in vivo* [51]–[55]. Inducers of apoptosis in epithelial cells include dsRNA signaling through PRRs, NS1, and NA. In our wd-NHBE model we observed a change in the epithelial cell layer structure after infection. We observed a loss of monolayer depth and desquamated cells as seen in previous models of infection [55]. We sought to explain this change in phenotype using our microarray data. We found factors, previously shown to alter the epithelial phenotype of the cell, are differentially regulated in KY/180 compared to the other isolates including KRT genes and those involved in cilia formation (FOXJ1, AKAP14, and PROM1) [39]. Furthermore, we looked at apoptosis pathways to determine whether these pathways were different between infections. We found that KY/180 and KY/136 significantly up-regulated BAK1 and Caspase 7, an apoptosis inducer, and down-regulated the CDK1 gene, a cell division control protein (Figures 8A–F, S2). This suggests any differences in phenotype, such as replication and cytokine and chemokine secretion, between isolates may be related to cellular integrity and state of differentiation.

Limited research is available providing a comprehensive gene expression profile of DEG in response to IAV infection of wd-NHBE cells. In a recent study conducted by Lee *et al*., on type I-like alveolar epithelial cells infected with H1N1pdm (A/Hong Kong/415742/2009) and seasonal H1N1 (A/Hong Kong/54/1998), 88 genes were found to be up or down-regulated in response to seasonal H1N1 infection while only 18 genes were affected in H1N1pdm infected cells [56]. IFN-induced genes, including IL28A, IL28B, IL29, IRF9, ISG15 and MX1, were significantly up-regulated in response to both H1N1pdm and seasonal H1N1 infections and to a similar degree. Additionally, Ioannidis *et al*. demonstrated that, in IAV infected primary differentiated lung epithelial cells, the most represented category of DEGs included the IFN-inducible genes, IFN-induced cytokines and chemokines, and PRRs [52]. Our data agree that both seasonal and pandemic isolates up-regulate IFN-induced genes; however, in our model, the degree of the response was greater in H1N1pdm infected cells compared to seasonal. We saw similar trends overall in terms of an elevated type-I IFN and antiviral responses, and additionally, we show a difference in genes involved in cellular differentiation.

In summary, we demonstrate the value of the wd-NHBE cell model in understanding the early events of viral infection, and unraveling clues to strain-specific, and pandemic versus seasonal virus-host interactions. Our studies provide preliminary evidence that strain specific differences between closely related pandemic viruses during infection of the lung epithelium may contribute to the trajectory of host responses and pathogenesis observed in mice and in humans [36]. By directly comparing pandemic and seasonal IAV isolates, we found unique differences in virus titer and cytokine and chemokine secretion between isolates. Intriguingly, there are only 22 amino acid mostly synonymous changes between KY/180 and KY/136 and of these only one of these so far in the HA (D222G) has been suggested to correlate with higher virulence in patients [57], [58]. Future studies will evaluate the role of the D222G and other amino acids in conferring the greater levels of virus, basal secretion of cytokines and apparent epithelial damage noted by KY/180. Further, future studies that couple *in vitro* human primary cell culture models with immune cells will be an important step in developing a fuller understanding the outcomes of viral-host interactions.

## Supporting Information

Figure S1 **Principle Component Analysis (PCA) for quality control of data.** Upon initial data analysis, log_2_ transformed expression intensity values were imported into Partek Genomic Suite software (V 6.5). We performed quality control with PCA analysis to ensure the three replicates per viral treatment grouped together. A plot of the first two components of the PCA (explaining 51.8% of the variation) showed that virus-infected isolates were different from mock-infected cells. Additionally both 2009 H1N1 IAV pandemic isolates (KY/180 and KY/136) clustered separately from the 2007 seasonal H1N1 IAV vaccine strain, BN/59.
(TIF)

Figure S2 **Apoptosis Signaling Pathway.** Ingenuity pathway analysis (genes whose expression changed by 2-fold with p<0.05 relative to mock infected control) showing the apoptosis canonical pathway after infection of wd-NHBE with (A) KY/180, (B) KY/136, and (C) BN/59 at 36 hpi. Different color intensities of ingenuity symbols indicate different levels of gene expression. Red indicates increased expression and green indicates decreased expression.
(TIF)

Figure S3 **ERK/MAPK Signaling Pathway.** Ingenuity pathway analysis (genes whose expression changed by 2-fold with p<0.05 relative to mock infected control) showing the ERK/MAPK canonical pathway after infection of wd-NHBE with (A) KY/180, (B) KY/136, and (C) BN/59 at 36hpi. Different color intensities of ingenuity symbols indicate different levels of gene expression. Red indicates increased expression and green indicates decreased expression.
(TIF)

Table S1 **GenBank accession numbers for isolates used in this study.**
(DOCX)

Table S2 **Significant cytokines and chemokines as determined by one-way ANOVA KY/180 compared to KY/136 (significant difference indicated at p<0.05).**
(DOCX)

Table S3 **Significant cytokines and chemokines as determined by one-way ANOVA: KY/180 compared to BN/59 (significant difference indicated with P<0.05).**
(DOCX)

Table S4 **Significant cytokines and chemokines as determined by one-way ANOVA: KY/136 compared to BN/59.**
(DOCX)

Table S5 **355 DEGs common to all three isolates at 36 hpi.**
(DOCX)

Table S6 **Differentially expressed genes unique to BN/59 infected wd-NHBE cells at 36 hpi.**
(DOCX)

Table S7 **Top 25 significantly differentially expressed genes unique to KY/180 infected wd-NHBE cells at 36 hpi.**
(DOCX)

Table S8 **Top 25 significantly differentially expressed genes unique to KY/136 infected wd-NHBE cells at 36 hpi.**
(DOCX)

Table S9 **Apoptosis genes differentially expressed at 36 hpi.**
(DOCX)
