# Supplementary figures and images for: Early Host Responses of Seasonal and Pandemic Influenza A Viruses in Primary Well-Differentiated Human Lung Epithelial Cells
Source: PLoS One. 2013 Nov 14;8(11):e78912. doi: 10.1371/journal.pone.0078912 (PMC3828299; doi:10.1371/journal.pone.0078912)

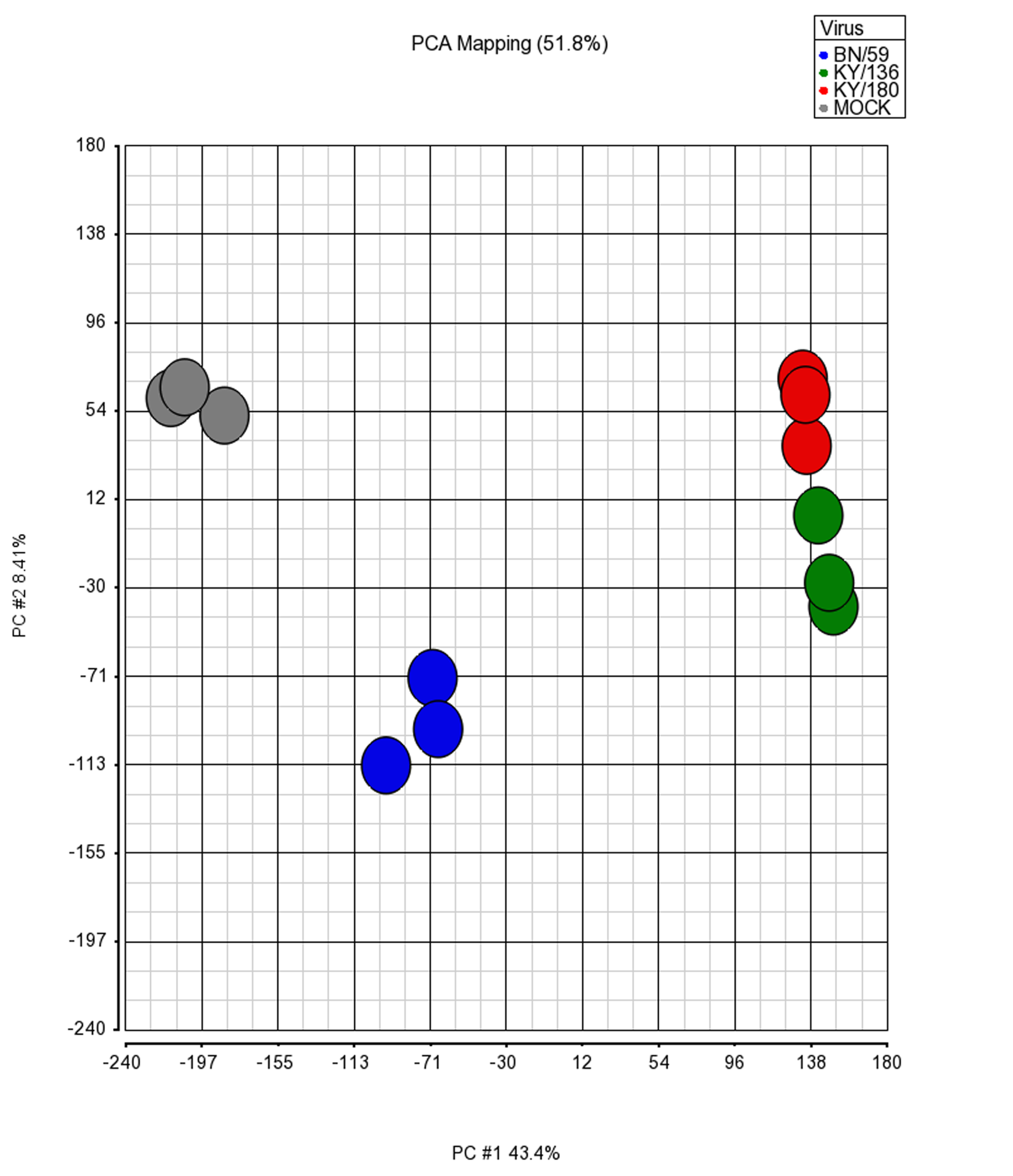

Supplement: Figure S1 — Principle Component Analysis (PCA) for quality control of data. Upon initial data analysis, log2 transformed expression intensity values were imported into Partek Genomic Suite software (V 6.5). We performed quality control with PCA analysis to ensure the three replicates per viral treatment grouped together. A plot of the first two components of the PCA (explaining 51.8% of the variation) showed that virus-infected isolates were different from mock-infected cells. Additionally both 2009 H1N1 IAV pandemic isolates (KY/180 and KY/136) clustered separately from the 2007 seasonal H1N1 IAV vaccine strain, BN/59. (TIF) [file pone.0078912.s001.tif]

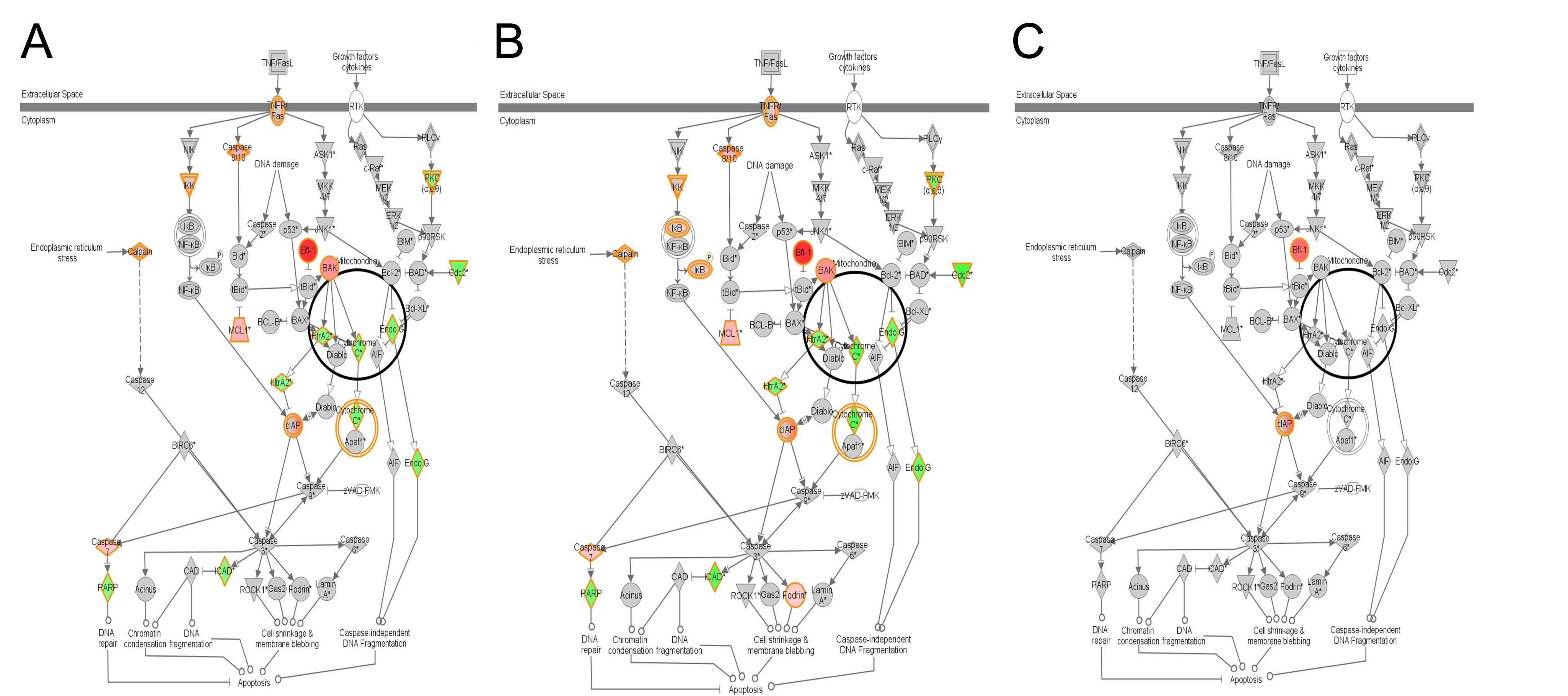

Supplement: Figure S2 — Apoptosis Signaling Pathway. Ingenuity pathway analysis (genes whose expression changed by 2-fold with p<0.05 relative to mock infected control) showing the apoptosis canonical pathway after infection of wd-NHBE with (A) KY/180, (B) KY/136, and (C) BN/59 at 36 hpi. Different color intensities of ingenuity symbols indicate different levels of gene expression. Red indicates increased expression and green indicates decreased expression. (TIF) [file pone.0078912.s002.tif]

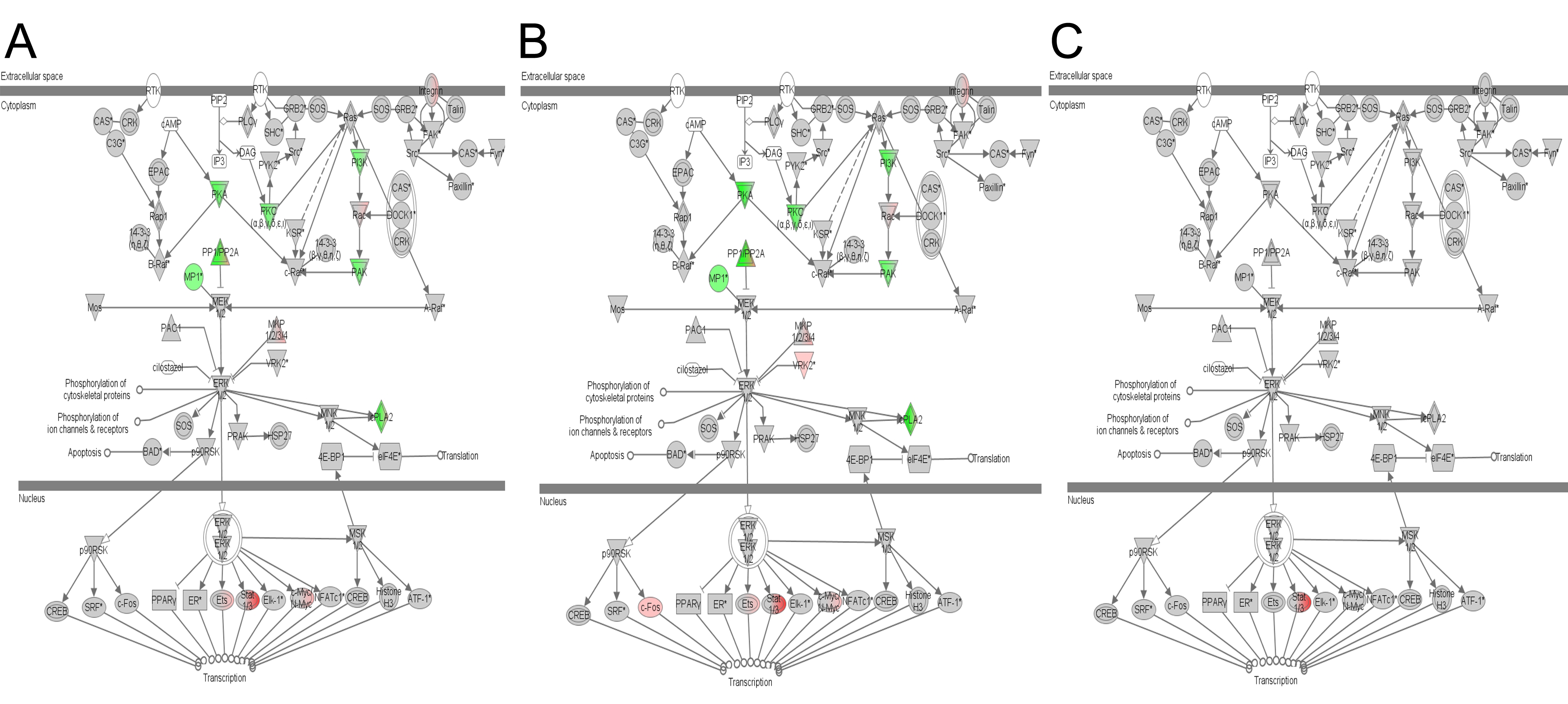

Supplement: Figure S3 — ERK/MAPK Signaling Pathway. Ingenuity pathway analysis (genes whose expression changed by 2-fold with p<0.05 relative to mock infected control) showing the ERK/MAPK canonical pathway after infection of wd-NHBE with (A) KY/180, (B) KY/136, and (C) BN/59 at 36hpi. Different color intensities of ingenuity symbols indicate different levels of gene expression. Red indicates increased expression and green indicates decreased expression. (TIF) [file pone.0078912.s003.tif]
