# Supplementary material for: Early Host Responses of Seasonal and Pandemic Influenza A Viruses in Primary Well-Differentiated Human Lung Epithelial Cells
Source: PLoS One. 2013 Nov 14;8(11):e78912. doi: 10.1371/journal.pone.0078912 (PMC3828299; doi:10.1371/journal.pone.0078912)
Supplement: Table S1 — GenBank accession numbers for isolates used in this study. (DOCX) [file pone.0078912.s004.docx]

**Table S1. GenBank accession numbers for isolates used in this study**

|  | **HA** | **M** | **NA** | **NP** | **NS** | **PA** | **PB1** | **PB2** |
| --- | --- | --- | --- | --- | --- | --- | --- | --- |
| **A/Kentucky/136/09/E** | CY099330 | JX875021 | CY099331 | JX875026 | JX875025 | JX875024 | JX875022 | JX875023 |
| **A/Kentucky/180/10/E** | CY099332 | JX875027 | CY099333 | JX875032 | JX875031 | JX875030 | JX875028 | JX875029 |
