# Supplementary material for: Early Host Responses of Seasonal and Pandemic Influenza A Viruses in Primary Well-Differentiated Human Lung Epithelial Cells
Source: PLoS One. 2013 Nov 14;8(11):e78912. doi: 10.1371/journal.pone.0078912 (PMC3828299; doi:10.1371/journal.pone.0078912)
Supplement: Table S2 — Significant cytokines and chemokines as determined by one-way ANOVA KY/180 compared to KY/136 (significant difference indicated at p<0.05). (DOCX) [file pone.0078912.s005.docx]

**Table S2. Significant cytokines and chemokines as determined by one-way ANOVA KY/180 compared to KY/136 (significant difference indicated at p<0.05).**

|  | APICAL | | | | | BASAL | | | | |
| --- | --- | --- | --- | --- | --- | --- | --- | --- | --- | --- |
|  | 8 | 24 | 36 | 48 | 72 | 8 | 24 | 36 | 48 | 72 |
| GM-CSF | NS | NS | NS | NS | NS | NS | NS | NS | NS | NS |
| G-CSF | NS | NS | NS | NS | NS | NS | NS | NS | NS | NS |
| GRO | NS | NS | NS | NS | NS | NS | NS | P<0.001 | NS | NS |
| IL6 | NS | NS | NS | NS | NS | NS | NS | P<0.001 | NS | P<0.05 |
| IL8 | NS | NS | NS | NS | NS | NS | NS | NS | NS | NS |
| MCP1 | NS | NS | NS | NS | NS | NS | NS | P<0.05 | NS | NS |
| CCL5 | NS | NS | P<0.01 | NS | NS | P<0.001 | NS | NS | NS | P<0.01 |
| IFNA2 | NS | NS | NS | NS | NS | NS | NS | NS | NS | NS |
| CXCL10 | NS | NS | NS | NS | NS | NS | NS | NS | NS | NS |
| TNFα | NS | P<0.05 | NS | NS | NS | NS | P<0.01 | NS | NS | NS |
| CCL4 | NS | NS | NS | NS | NS | NS | NS | NS | P<0.001 | P<0.05 |
| IL10 | NS | NS | NS | NS | NS | NS | NS | NS | NS | NS |
