# Supplementary material for: Early Host Responses of Seasonal and Pandemic Influenza A Viruses in Primary Well-Differentiated Human Lung Epithelial Cells
Source: PLoS One. 2013 Nov 14;8(11):e78912. doi: 10.1371/journal.pone.0078912 (PMC3828299; doi:10.1371/journal.pone.0078912)
Supplement: Table S5 — 355 DEGs common to all three isolates at 36 hpi. (DOCX) [file pone.0078912.s008.docx]

**Table S5. 355 DEGs common to all three isolates at 36 hpi**

| **Symbol** | **Entrez Gene Name** | **Affymetrix Probe** | **Fold Change KY/180** | **Fold Change KY/136** | **Fold Change BN/59** |
| --- | --- | --- | --- | --- | --- |
| CXCL11 | chemokine (C-X-C motif) ligand 11 | 211122_s_at | 150.026 | 147.372 | 86.740 |
| CXCL10 | chemokine (C-X-C motif) ligand 10 | 204533_at | 127.355 | 128.617 | 77.292 |
| OASL | 2'-5'-oligoadenylate synthetase-like | 205660_at | 59.701 | 67.739 | 22.999 |
| IFIT2 | interferon-induced protein with tetratricopeptide repeats 2 | 217502_at | 59.648 | 66.056 | 31.475 |
| TNFSF13B | tumor necrosis factor (ligand) superfamily, member 13b | 223502_s_at | 50.991 | 58.491 | 21.951 |
| DUOXA2 | dual oxidase maturation factor 2 | 230615_at | 35.278 | 42.264 | 9.158 |
| RSAD2 | radical S-adenosyl methionine domain containing 2 | 242625_at | 37.866 | 37.897 | 26.356 |
| IFIT3 | interferon-induced protein with tetratricopeptide repeats 3 | 204747_at | 27.807 | 29.105 | 18.812 |
| AIM2 | absent in melanoma 2 | 206513_at | 23.594 | 26.998 | 6.573 |
| GMPR | guanosine monophosphate reductase | 204187_at | 22.314 | 26.249 | 9.796 |
| IL28A | interleukin 28A (interferon, lambda 2) | 1552609_s_at | 16.088 | 26.224 | 4.019 |
| IFIT1 | interferon-induced protein with tetratricopeptide repeats 1 | 203153_at | 25.481 | 25.278 | 22.239 |
| BATF2 | basic leucine zipper transcription factor, ATF-like 2 | 228439_at | 19.459 | 24.155 | 7.861 |
| IFITM1 | interferon induced transmembrane protein 1 | 201601_x_at | 23.577 | 23.781 | 19.370 |
| HERC5 | HECT and RLD domain containing E3 ubiquitin protein ligase 5 | 219863_at | 21.616 | 23.631 | 16.598 |
| MX2 | myxovirus (influenza virus) resistance 2 (mouse) | 204994_at | 22.346 | 23.548 | 18.551 |
| DDX58 | DEAD (Asp-Glu-Ala-Asp) box polypeptide 58 | 218943_s_at | 21.423 | 23.252 | 12.166 |
| EPSTI1 | epithelial stromal interaction 1 (breast) | 227609_at | 21.435 | 22.679 | 14.101 |
| ISG15 | ISG15 ubiquitin-like modifier | 205483_s_at | 21.858 | 21.977 | 17.955 |
| CD38 | CD38 molecule | 205692_s_at | 14.473 | 21.593 | 6.665 |
| IL4I1 | interleukin 4 induced 1 | 230966_at | 15.246 | 21.072 | 2.882 |
| NEXN | nexilin (F actin binding protein) | 1552309_a_at | 13.716 | 20.840 | 4.763 |
| APOBEC3A | apolipoprotein B mRNA editing enzyme, catalytic polypeptide-like 3A | 210873_x_at | 21.187 | 20.484 | 8.119 |
| IDO1 | indoleamine 2,3-dioxygenase 1 | 210029_at | 17.614 | 19.363 | 9.826 |
| BST2 | bone marrow stromal cell antigen 2 | 201641_at | 18.705 | 19.301 | 12.451 |
| CCL5 | chemokine (C-C motif) ligand 5 | 1555759_a_at | 15.058 | 19.064 | 3.449 |
| MX1 | myxovirus (influenza virus) resistance 1, interferon-inducible protein p78 (mouse) | 202086_at | 17.521 | 18.425 | 14.804 |
| BRIP1 | BRCA1 interacting protein C-terminal helicase 1 | 235609_at | 14.288 | 17.615 | 4.472 |
| ARL14 | ADP-ribosylation factor-like 14 | 220468_at | 15.026 | 17.297 | 3.624 |
| IFI35 | interferon-induced protein 35 | 209417_s_at | 16.300 | 17.240 | 8.669 |
| CMPK2 | cytidine monophosphate (UMP-CMP) kinase 2, mitochondrial | 226702_at | 16.155 | 16.776 | 12.895 |
| IL29 | interleukin 29 (interferon, lambda 1) | 1552917_at | 9.911 | 16.125 | 2.919 |
| SAMD9L | sterile alpha motif domain containing 9-like | 226603_at | 15.462 | 16.108 | 8.699 |
| CEACAM1 | carcinoembryonic antigen-related cell adhesion molecule 1 (biliary glycoprotein) | 211883_x_at | 15.137 | 15.823 | 3.905 |
| FRMD3 | FERM domain containing 3 | 230645_at | 12.669 | 15.821 | 6.623 |
| IFI44L | interferon-induced protein 44-like | 204439_at | 15.811 | 15.755 | 14.875 |
| ISG20 | interferon stimulated exonuclease gene 20kDa | 204698_at | 14.265 | 15.726 | 9.254 |
| XAF1 | XIAP associated factor 1 | 228617_at | 14.765 | 15.438 | 9.719 |
| CD274 | CD274 molecule | 227458_at | 11.640 | 15.275 | 4.252 |
| USP18 | ubiquitin specific peptidase 18 | 219211_at | 13.571 | 15.148 | 8.690 |
| GBP5 | guanylate binding protein 5 | 229625_at | 11.390 | 14.519 | 3.728 |
| ZBP1 | Z-DNA binding protein 1 | 208087_s_at | 11.722 | 14.293 | 4.620 |
| HTR2B | 5-hydroxytryptamine (serotonin) receptor 2B, G protein-coupled | 206638_at | 10.219 | 13.869 | 3.441 |
| MMP13 | matrix metallopeptidase 13 (collagenase 3) | 205959_at | 11.564 | 13.862 | 3.998 |
| HRASLS2 | HRAS-like suppressor 2 | 221122_at | 12.398 | 13.828 | 6.241 |
| SOCS1 | suppressor of cytokine signaling 1 | 210001_s_at | 9.705 | 13.695 | 5.296 |
| TAP1 | transporter 1, ATP-binding cassette, sub-family B (MDR/TAP) | 202307_s_at | 12.560 | 13.475 | 5.940 |
| OAS3 | 2'-5'-oligoadenylate synthetase 3, 100kDa | 218400_at | 13.267 | 13.433 | 10.660 |
| TMEM140 | transmembrane protein 140 | 218999_at | 10.922 | 13.217 | 5.999 |
| SLC15A3 | solute carrier family 15, member 3 | 219593_at | 12.261 | 13.055 | 4.845 |
| OAS2 | 2'-5'-oligoadenylate synthetase 2, 69/71kDa | 204972_at | 12.815 | 12.871 | 11.853 |
| OAS1 | 2'-5'-oligoadenylate synthetase 1, 40/46kDa | 205552_s_at | 12.208 | 12.560 | 7.482 |
| STAT1 | signal transducer and activator of transcription 1, 91kDa | 209969_s_at | 12.366 | 12.505 | 9.504 |
| GBP4 | guanylate binding protein 4 | 235574_at | 11.476 | 12.473 | 5.367 |
| BCL2A1 | BCL2-related protein A1 | 205681_at | 11.290 | 12.048 | 2.574 |
| IFIH1 | interferon induced with helicase C domain 1 | 219209_at | 11.712 | 12.026 | 7.725 |
| GBP1 | guanylate binding protein 1, interferon-inducible | 231577_s_at | 10.839 | 11.934 | 6.859 |
| KLHDC7B | kelch domain containing 7B | 236285_at | 10.758 | 11.899 | 5.258 |
| SERPING1 | serpin peptidase inhibitor, clade G (C1 inhibitor), member 1 | 200986_at | 9.444 | 11.852 | 4.480 |
| ETV7 | ets variant 7 | 224225_s_at | 9.914 | 11.828 | 6.384 |
| NCF1C | neutrophil cytosolic factor 1C pseudogene | 214084_x_at | 10.269 | 11.697 | 2.169 |
| DFNB31 | deafness, autosomal recessive 31 | 221887_s_at | 10.714 | 11.499 | 8.218 |
| LMO2 | LIM domain only 2 (rhombotin-like 1) | 204249_s_at | 9.163 | 11.388 | 4.382 |
| THEMIS2 | thymocyte selection associated family member 2 | 210785_s_at | 9.199 | 11.338 | 3.722 |
| LAG3 | lymphocyte-activation gene 3 | 206486_at | 7.409 | 11.063 | 2.855 |
| TRAC | T cell receptor alpha constant | 215524_x_at | 10.090 | 10.966 | 4.807 |
| HIST1H3A (includes others) | histone cluster 1, H3a | 214472_at | 11.015 | 10.889 | 3.023 |
| WARS | tryptophanyl-tRNA synthetase | 200628_s_at | 10.520 | 10.855 | 3.976 |
| TAGAP | T-cell activation RhoGTPase activating protein | 229723_at | 9.810 | 10.839 | 3.317 |
| SP110 | SP110 nuclear body protein | 223980_s_at | 10.241 | 10.477 | 5.935 |
| NUPR1 | nuclear protein, transcriptional regulator, 1 | 209230_s_at | 9.509 | 10.409 | 5.530 |
| CLEC7A | C-type lectin domain family 7, member A | 221698_s_at | 8.680 | 10.323 | 3.827 |
| NLRC5 | NLR family, CARD domain containing 5 | 226474_at | 9.056 | 10.315 | 4.190 |
| HESX1 | HESX homeobox 1 | 211267_at | 6.133 | 10.178 | 3.038 |
| FGD2 | FYVE, RhoGEF and PH domain containing 2 | 1553906_s_at | 8.278 | 10.124 | 2.870 |
| IRF7 | interferon regulatory factor 7 | 208436_s_at | 8.989 | 9.978 | 5.466 |
| DDX60L | DEAD (Asp-Glu-Ala-Asp) box polypeptide 60-like | 228152_s_at | 10.552 | 9.895 | 7.858 |
| LAMP3 | lysosomal-associated membrane protein 3 | 205569_at | 9.651 | 9.867 | 6.612 |
| NPBWR1 | neuropeptides B/W receptor 1 | 231752_at | 9.405 | 9.283 | 4.814 |
| CD68 | CD68 molecule | 203507_at | 7.836 | 9.196 | 2.593 |
| IFITM2 | interferon induced transmembrane protein 2 | 201315_x_at | 8.771 | 9.130 | 6.460 |
| DUOX2 | dual oxidase 2 | 219727_at | 9.043 | 8.925 | 4.915 |
| C1S | complement component 1, s subcomponent | 208747_s_at | 8.362 | 8.852 | 2.909 |
| LOC100507025 | -- | 232035_at | 7.668 | 8.799 | 2.163 |
| BCL2L14 | BCL2-like 14 (apoptosis facilitator) | 221241_s_at | 6.845 | 8.734 | 4.246 |
| HIST1H4A (includes others) | histone cluster 1, H4a | 208180_s_at | 7.469 | 8.690 | 2.121 |
| HSH2D | hematopoietic SH2 domain containing | 1552623_at | 7.650 | 8.646 | 4.005 |
| MLKL | mixed lineage kinase domain-like | 238025_at | 7.241 | 8.632 | 3.109 |
| LIFR | leukemia inhibitory factor receptor alpha | 225575_at | 6.076 | 8.331 | 4.172 |
| ERAP2 | endoplasmic reticulum aminopeptidase 2 | 227462_at | 7.843 | 8.314 | 3.004 |
| ZC3HAV1 | zinc finger CCCH-type, antiviral 1 | 220104_at | 7.059 | 8.294 | 3.773 |
| RASGRP3 | RAS guanyl releasing protein 3 (calcium and DAG-regulated) | 205801_s_at | 6.848 | 8.230 | 2.915 |
| APOBEC3G | apolipoprotein B mRNA editing enzyme, catalytic polypeptide-like 3G | 204205_at | 7.608 | 8.222 | 5.056 |
| SAMD9 | sterile alpha motif domain containing 9 | 219691_at | 8.123 | 8.196 | 4.401 |
| TOR1B | torsin family 1, member B (torsin B) | 209593_s_at | 7.315 | 8.196 | 3.869 |
| C19orf66 | chromosome 19 open reading frame 66 | 53720_at | 7.531 | 8.180 | 4.499 |
| NT5C3 | 5'-nucleotidase, cytosolic III | 223298_s_at | 8.082 | 8.170 | 5.534 |
| USP30-AS1 | USP30 antisense RNA 1 | 228675_at | 6.467 | 8.089 | 3.523 |
| LDB2 | LIM domain binding 2 | 242360_at | 7.743 | 8.035 | 3.389 |
| IFI44 | interferon-induced protein 44 | 214453_s_at | 7.625 | 8.031 | 6.822 |
| TNFRSF6B | tumor necrosis factor receptor superfamily, member 6b, decoy | 206467_x_at | 6.751 | 7.814 | 2.590 |
| PLAUR | plasminogen activator, urokinase receptor | 214866_at | 6.556 | 7.795 | 2.396 |
| RTP4 | receptor (chemosensory) transporter protein 4 | 219684_at | 7.089 | 7.727 | 5.546 |
| TRIM69 | tripartite motif containing 69 | 1568592_at | 7.273 | 7.695 | 4.287 |
| IL1RN | interleukin 1 receptor antagonist | 216243_s_at | 6.929 | 7.519 | 2.430 |
| HERC6 | HECT and RLD domain containing E3 ubiquitin protein ligase family member 6 | 219352_at | 7.117 | 7.443 | 6.069 |
| TRIM38 | tripartite motif containing 38 | 203610_s_at | 6.728 | 7.441 | 2.786 |
| TAP2 | transporter 2, ATP-binding cassette, sub-family B (MDR/TAP) | 204769_s_at | 6.775 | 7.433 | 4.240 |
| ACE2 | angiotensin I converting enzyme (peptidyl-dipeptidase A) 2 | 219962_at | 6.146 | 7.349 | 3.336 |
| VNN3 | vanin 3 | 220528_at | 7.204 | 7.322 | 2.828 |
| MXD1 | MAX dimerization protein 1 | 228846_at | 6.796 | 7.122 | 2.317 |
| AKAP2/PALM2-AKAP2 | A kinase (PRKA) anchor protein 2 | 226694_at | 5.501 | 7.117 | 3.131 |
| TRIM21 | tripartite motif containing 21 | 204804_at | 6.098 | 7.110 | 3.381 |
| NEURL3 | neuralized homolog 3 (Drosophila) pseudogene | 232593_at | 5.602 | 7.100 | 2.175 |
| CLEC2B | C-type lectin domain family 2, member B | 209732_at | 5.950 | 7.091 | 3.203 |
| SLC25A28 | solute carrier family 25 (mitochondrial iron transporter), member 28 | 221432_s_at | 6.157 | 7.083 | 3.511 |
| SELL | selectin L | 204563_at | 6.173 | 6.952 | 2.672 |
| FAM46A | family with sequence similarity 46, member A | 221766_s_at | 6.483 | 6.907 | 2.996 |
| PML | promyelocytic leukemia | 209640_at | 6.161 | 6.886 | 3.918 |
| IFI6 | interferon, alpha-inducible protein 6 | 204415_at | 6.811 | 6.817 | 6.335 |
| ELOVL7 | ELOVL fatty acid elongase 7 | 227180_at | 5.676 | 6.809 | 2.448 |
| GSDMB | gasdermin B | 219233_s_at | 5.661 | 6.738 | 2.468 |
| FAM125A | family with sequence similarity 125, member A | 1556643_at | 5.009 | 6.723 | 4.191 |
| PARP9 | poly (ADP-ribose) polymerase family, member 9 | 223220_s_at | 6.232 | 6.677 | 4.882 |
| MGC39372 | serpin peptidase inhibitor, clade B (ovalbumin), member 9 pseudogene | 239186_at | 5.080 | 6.672 | 2.209 |
| PRNP | prion protein | 1556190_s_at | 5.623 | 6.670 | 2.122 |
| LGALS9 | lectin, galactoside-binding, soluble, 9 | 203236_s_at | 5.824 | 6.628 | 2.918 |
| APOL2 | apolipoprotein L, 2 | 221653_x_at | 6.201 | 6.563 | 3.256 |
| UBE2L6 | ubiquitin-conjugating enzyme E2L 6 | 201649_at | 6.065 | 6.552 | 5.072 |
| PNLIPRP3 | pancreatic lipase-related protein 3 | 1558846_at | 6.651 | 6.535 | 2.052 |
| PARP12 | poly (ADP-ribose) polymerase family, member 12 | 218543_s_at | 5.935 | 6.502 | 4.376 |
| C1R | complement component 1, r subcomponent | 212067_s_at | 6.311 | 6.498 | 2.778 |
| PPM1K | protein phosphatase, Mg2+/Mn2+ dependent, 1K | 235061_at | 5.588 | 6.443 | 4.382 |
| IL18BP | interleukin 18 binding protein | 222868_s_at | 4.976 | 6.391 | 2.300 |
| RBM11 | RNA binding motif protein 11 | 232549_at | 4.611 | 6.366 | 2.630 |
| TLR3 | toll-like receptor 3 | 206271_at | 5.907 | 6.325 | 3.427 |
| PSMB9 | proteasome (prosome, macropain) subunit, beta type, 9 (large multifunctional peptidase 2) | 204279_at | 5.624 | 6.315 | 3.954 |
| TMEM171 | transmembrane protein 171 | 240770_at | 5.027 | 6.289 | 2.292 |
| APOL1 | apolipoprotein L, 1 | 209546_s_at | 6.367 | 6.202 | 3.501 |
| SLC16A4 | solute carrier family 16, member 4 (monocarboxylic acid transporter 5) | 205234_at | 5.723 | 6.121 | 2.214 |
| LY6E | lymphocyte antigen 6 complex, locus E | 202145_at | 5.683 | 6.098 | 3.725 |
| SOCS2 | suppressor of cytokine signaling 2 | 203373_at | 5.729 | 6.080 | 2.053 |
| BIRC3 | baculoviral IAP repeat containing 3 | 210538_s_at | 5.422 | 6.071 | 3.121 |
| DDX60 | DEAD (Asp-Glu-Ala-Asp) box polypeptide 60 | 218986_s_at | 6.780 | 6.033 | 6.109 |
| TYMP | thymidine phosphorylase | 204858_s_at | 6.402 | 6.031 | 3.270 |
| APOL3 | apolipoprotein L, 3 | 221087_s_at | 5.224 | 5.993 | 3.110 |
| C9orf84 | chromosome 9 open reading frame 84 | 233504_at | 5.766 | 5.957 | 2.115 |
| XDH | xanthine dehydrogenase | 241994_at | 6.156 | 5.951 | 2.266 |
| PNPT1 | polyribonucleotide nucleotidyltransferase 1 | 225291_at | 5.287 | 5.916 | 3.718 |
| IL15RA | interleukin 15 receptor, alpha | 207375_s_at | 4.946 | 5.883 | 2.709 |
| C5orf56 | chromosome 5 open reading frame 56 | 230405_at | 4.823 | 5.875 | 3.193 |
| TLR2 | toll-like receptor 2 | 204924_at | 5.396 | 5.874 | 2.609 |
| HCP5 | HLA complex P5 (non-protein coding) | 206082_at | 5.456 | 5.771 | 3.243 |
| TRIB2 | tribbles homolog 2 (Drosophila) | 202478_at | 5.211 | 5.727 | 2.542 |
| ERICH1-AS1 | ERICH1 antisense RNA 1 | 1557679_at | 5.652 | 5.720 | 2.523 |
| ZNFX1 | zinc finger, NFX1-type containing 1 | 225076_s_at | 5.106 | 5.700 | 3.314 |
| MICB | MHC class I polypeptide-related sequence B | 206247_at | 3.864 | 5.692 | 2.461 |
| CCRL1 | chemokine (C-C motif) receptor-like 1 | 220351_at | 4.930 | 5.680 | 2.685 |
| APOL6 | apolipoprotein L, 6 | 219716_at | 5.282 | 5.678 | 3.165 |
| JAK2 | Janus kinase 2 | 205842_s_at | 4.936 | 5.646 | 2.277 |
| CSRNP1 | cysteine-serine-rich nuclear protein 1 | 225557_at | 4.679 | 5.585 | 2.645 |
| GIMAP2 | GTPase, IMAP family member 2 | 232024_at | 4.536 | 5.553 | 3.152 |
| HLA-F | major histocompatibility complex, class I, F | 221875_x_at | 5.278 | 5.546 | 3.455 |
| RBMS2 | RNA binding motif, single stranded interacting protein 2 | 34187_at | 4.874 | 5.472 | 2.285 |
| MT1M | metallothionein 1M | 217546_at | 3.371 | 5.465 | 2.273 |
| SP140 | SP140 nuclear body protein | 207777_s_at | 4.105 | 5.449 | 2.053 |
| CX3CL1 | chemokine (C-X3-C motif) ligand 1 | 823_at | 4.571 | 5.445 | 2.980 |
| PRIC285 | peroxisomal proliferator-activated receptor A interacting complex 285 | 228230_at | 5.205 | 5.443 | 3.332 |
| HLA-E | major histocompatibility complex, class I, E | 200904_at | 4.795 | 5.417 | 3.122 |
| SECTM1 | secreted and transmembrane 1 | 213716_s_at | 5.183 | 5.366 | 3.272 |
| MASTL | microtubule associated serine/threonine kinase-like | 228468_at | 4.845 | 5.345 | 2.606 |
| C8orf4 | chromosome 8 open reading frame 4 | 218541_s_at | 4.474 | 5.310 | 2.393 |
| STARD5 | StAR-related lipid transfer (START) domain containing 5 | 213820_s_at | 4.808 | 5.283 | 2.290 |
| HAPLN3 | hyaluronan and proteoglycan link protein 3 | 227262_at | 5.104 | 5.210 | 2.656 |
| APOB | apolipoprotein B (including Ag(x) antigen) | 205108_s_at | 5.278 | 5.191 | 2.417 |
| DHX58 | DEXH (Asp-Glu-X-His) box polypeptide 58 | 219364_at | 4.655 | 5.187 | 3.452 |
| LOC100506831 | -- | 237105_at | 4.356 | 5.167 | 2.633 |
| CASP1 | caspase 1, apoptosis-related cysteine peptidase | 211367_s_at | 4.686 | 5.156 | 2.710 |
| IFI30 | interferon, gamma-inducible protein 30 | 201422_at | 4.483 | 5.135 | 2.654 |
| HK2 | hexokinase 2 | 202934_at | 4.792 | 5.101 | 2.324 |
| RNF114 | ring finger protein 114 | 200868_s_at | 4.410 | 5.091 | 2.254 |
| TGM2 | transglutaminase 2 (C polypeptide, protein-glutamine-gamma-glutamyltransferase) | 201042_at | 4.246 | 5.090 | 3.322 |
| SLAMF9 | SLAM family member 9 | 1553769_at | 4.506 | 5.088 | 3.096 |
| IRF1 | interferon regulatory factor 1 | 238725_at | 4.177 | 5.088 | 3.092 |
| PHLPP2 | PH domain and leucine rich repeat protein phosphatase 2 | 213407_at | 4.040 | 5.076 | 2.999 |
| PARP10 | poly (ADP-ribose) polymerase family, member 10 | 229350_x_at | 4.601 | 5.063 | 3.622 |
| PCGF5 | polycomb group ring finger 5 | 227935_s_at | 4.718 | 5.040 | 2.404 |
| TRIM5 | tripartite motif containing 5 | 210705_s_at | 5.051 | 5.036 | 3.216 |
| CYP2J2 | cytochrome P450, family 2, subfamily J, polypeptide 2 | 205073_at | 4.282 | 4.994 | 2.513 |
| TRANK1 | tetratricopeptide repeat and ankyrin repeat containing 1 | 213261_at | 4.744 | 4.982 | 3.232 |
| EMP1 | epithelial membrane protein 1 | 201325_s_at | 4.643 | 4.937 | 2.131 |
| EML2 | echinoderm microtubule associated protein like 2 | 234021_at | 4.407 | 4.916 | 2.160 |
| IL8 | interleukin 8 | 211506_s_at | 4.992 | 4.863 | 2.654 |
| MUC13 | mucin 13, cell surface associated | 222712_s_at | 4.218 | 4.862 | 2.559 |
| MGAT3 | mannosyl (beta-1,4-)-glycoprotein beta-1,4-N-acetylglucosaminyltransferase | 209764_at | 3.869 | 4.839 | 2.001 |
| ADRB2 | adrenoceptor beta 2, surface | 206170_at | 4.063 | 4.819 | 2.250 |
| NMI | N-myc (and STAT) interactor | 203964_at | 4.679 | 4.813 | 3.562 |
| LGMN | legumain | 201212_at | 4.461 | 4.811 | 2.241 |
| HBEGF | heparin-binding EGF-like growth factor | 203821_at | 4.494 | 4.778 | 2.101 |
| SEC16B | SEC16 homolog B (S. cerevisiae) | 228150_at | 4.091 | 4.761 | 2.481 |
| GLRX | glutaredoxin (thioltransferase) | 209276_s_at | 4.347 | 4.759 | 2.240 |
| BTN3A3 | butyrophilin, subfamily 3, member A3 | 204821_at | 4.269 | 4.724 | 3.150 |
| LPAR6 | lysophosphatidic acid receptor 6 | 218589_at | 4.126 | 4.722 | 2.153 |
| PRKRA | protein kinase, interferon-inducible double stranded RNA dependent activator | 228714_at | 4.217 | 4.710 | 3.317 |
| RNF213 | ring finger protein 213 | 225929_s_at | 4.866 | 4.622 | 3.539 |
| GBP1P1 | guanylate binding protein 1, interferon-inducible pseudogene 1 | 1570541_s_at | 4.160 | 4.621 | 2.072 |
| ADAM28 | ADAM metallopeptidase domain 28 | 205997_at | 4.166 | 4.590 | 2.257 |
| LOC100288123 | uncharacterized LOC100288123 | 230940_at | 4.691 | 4.534 | 3.282 |
| IFITM3 | interferon induced transmembrane protein 3 | 212203_x_at | 4.497 | 4.506 | 4.208 |
| NUB1 | negative regulator of ubiquitin-like proteins 1 | 222512_at | 3.972 | 4.482 | 2.858 |
| DUSP5 | dual specificity phosphatase 5 | 209457_at | 3.940 | 4.458 | 2.035 |
| BLZF1 | basic leucine zipper nuclear factor 1 | 32088_at | 4.330 | 4.443 | 2.344 |
| STARD4 | StAR-related lipid transfer (START) domain containing 4 | 226390_at | 4.192 | 4.434 | 2.282 |
| GCH1 | GTP cyclohydrolase 1 | 204224_s_at | 3.868 | 4.427 | 2.193 |
| MDK | midkine (neurite growth-promoting factor 2) | 209035_at | 3.768 | 4.337 | 2.318 |
| HLA-A | major histocompatibility complex, class I, A | 217436_x_at | 4.021 | 4.299 | 2.596 |
| RHBDD1 | rhomboid domain containing 1 | 233164_x_at | 4.521 | 4.291 | 3.001 |
| TNFSF13 | tumor necrosis factor (ligand) superfamily, member 13 | 210314_x_at | 3.502 | 4.280 | 2.366 |
| STAT2 | signal transducer and activator of transcription 2, 113kDa | 225636_at | 3.935 | 4.272 | 3.141 |
| CFLAR | CASP8 and FADD-like apoptosis regulator | 209939_x_at | 3.914 | 4.267 | 2.022 |
| EHD4 | EH-domain containing 4 | 229074_at | 3.715 | 4.265 | 2.443 |
| TREX1 | three prime repair exonuclease 1 | 205875_s_at | 3.253 | 4.239 | 2.482 |
| FBXO6 | F-box protein 6 | 231769_at | 3.733 | 4.210 | 2.299 |
| GCA | grancalcin, EF-hand calcium binding protein | 203765_at | 3.649 | 4.210 | 2.061 |
| KIAA0226L | KIAA0226-like | 219471_at | 4.126 | 4.208 | 2.277 |
| FBXW12 | F-box and WD repeat domain containing 12 | 1564138_at | 4.291 | 4.180 | 2.492 |
| RIF1 | RAP1 interacting factor homolog (yeast) | 214872_at | 3.713 | 4.173 | 2.808 |
| LOC728769 | uncharacterized LOC728769 | 238039_at | 3.128 | 4.170 | 2.550 |
| SP140L | SP140 nuclear body protein-like | 214791_at | 3.448 | 4.167 | 2.059 |
| CARD16 | caspase recruitment domain family, member 16 | 1552701_a_at | 3.679 | 4.160 | 2.016 |
| ZNF618 | zinc finger protein 618 | 226590_at | 3.948 | 4.154 | 2.330 |
| FTSJD2 | FtsJ methyltransferase domain containing 2 | 212380_at | 3.931 | 4.144 | 2.751 |
| TRAFD1 | TRAF-type zinc finger domain containing 1 | 35254_at | 3.603 | 4.137 | 2.211 |
| NRCAM | neuronal cell adhesion molecule | 216959_x_at | 4.113 | 4.131 | 2.560 |
| APOL4 | apolipoprotein L, 4 | 1555600_s_at | 3.205 | 4.110 | 2.071 |
| CNP | 2',3'-cyclic nucleotide 3' phosphodiesterase | 208912_s_at | 3.732 | 4.103 | 2.366 |
| PRB1/PRB3 | proline-rich protein BstNI subfamily 1 | 211531_x_at | 4.218 | 4.082 | 2.693 |
| DTX3L | deltex 3-like (Drosophila) | 225415_at | 4.067 | 4.076 | 3.375 |
| TNFSF10 | tumor necrosis factor (ligand) superfamily, member 10 | 202688_at | 3.786 | 4.061 | 3.164 |
| CCDC109B | coiled-coil domain containing 109B | 218802_at | 3.523 | 4.034 | 2.045 |
| TDRD7 | tudor domain containing 7 | 213361_at | 3.423 | 4.025 | 2.780 |
| MICAL2 | microtubule associated monoxygenase, calponin and LIM domain containing 2 | 206275_s_at | 4.175 | 4.001 | 2.204 |
| IL1A | interleukin 1, alpha | 210118_s_at | 4.323 | 3.977 | 2.002 |
| SIDT1 | SID1 transmembrane family, member 1 | 219734_at | 3.352 | 3.974 | 2.408 |
| CABP4 | calcium binding protein 4 | 1554201_at | 4.025 | 3.965 | 3.025 |
| HIST2H2BE (includes others) | histone cluster 2, H2be | 202708_s_at | 3.489 | 3.953 | 2.070 |
| PHF15 | PHD finger protein 15 | 212660_at | 3.468 | 3.953 | 2.389 |
| PIK3AP1 | phosphoinositide-3-kinase adaptor protein 1 | 226459_at | 2.521 | 3.953 | 2.991 |
| DDIT3 | DNA-damage-inducible transcript 3 | 209383_at | 4.258 | 3.938 | 2.193 |
| ZFYVE26 | zinc finger, FYVE domain containing 26 | 213073_at | 3.608 | 3.897 | 2.505 |
| HLA-G | major histocompatibility complex, class I, G | 210514_x_at | 3.560 | 3.885 | 2.422 |
| PLEKHA4 | pleckstrin homology domain containing, family A (phosphoinositide binding specific) member 4 | 219011_at | 3.392 | 3.845 | 2.411 |
| IFI27 | interferon, alpha-inducible protein 27 | 202411_at | 3.926 | 3.816 | 3.575 |
| UCKL1 | uridine-cytidine kinase 1-like 1 | 232727_at | 3.938 | 3.796 | 2.118 |
| C4B (includes others) | complement component 4B (Chido blood group) | 214428_x_at | 2.682 | 3.774 | 2.571 |
| OPTN | optineurin | 202074_s_at | 3.337 | 3.761 | 2.507 |
| PARP14 | poly (ADP-ribose) polymerase family, member 14 | 224701_at | 3.667 | 3.739 | 3.313 |
| XRN1 | 5'-3' exoribonuclease 1 | 1555785_a_at | 3.668 | 3.702 | 2.536 |
| C20orf181 | chromosome 20 open reading frame 181 | 1567035_at | 3.671 | 3.699 | 2.425 |
| C10orf71 | chromosome 10 open reading frame 71 | 232456_at | 3.718 | 3.697 | 3.437 |
| GTPBP2 | GTP binding protein 2 | 221050_s_at | 3.532 | 3.696 | 2.098 |
| ACSL6 | acyl-CoA synthetase long-chain family member 6 | 216409_at | 3.963 | 3.694 | 3.031 |
| TRIM14 | tripartite motif containing 14 | 203148_s_at | 3.676 | 3.693 | 3.316 |
| AKAP7 | A kinase (PRKA) anchor protein 7 | 205771_s_at | 3.045 | 3.687 | 2.409 |
| PSMB8 | proteasome (prosome, macropain) subunit, beta type, 8 (large multifunctional peptidase 7) | 209040_s_at | 3.341 | 3.681 | 2.760 |
| HLA-C | major histocompatibility complex, class I, C | 211799_x_at | 3.564 | 3.677 | 2.558 |
| TNFAIP3 | tumor necrosis factor, alpha-induced protein 3 | 202643_s_at | 3.211 | 3.664 | 2.026 |
| SLC16A1 | solute carrier family 16, member 1 (monocarboxylic acid transporter 1) | 202236_s_at | 3.571 | 3.659 | 2.134 |
| MYD88 | myeloid differentiation primary response gene (88) | 209124_at | 3.539 | 3.644 | 2.298 |
| SCIN | scinderin | 1552365_at | 3.018 | 3.609 | 2.028 |
| LGALS3BP | lectin, galactoside-binding, soluble, 3 binding protein | 200923_at | 3.469 | 3.588 | 2.816 |
| IFI16 | IFNy-inducible protein 16 | 208965_s_at | 3.496 | 3.580 | 2.344 |
| MAFF | v-maf musculoaponeurotic fibrosarcoma oncogene homolog F (avian) | 36711_at | 3.242 | 3.548 | 2.181 |
| BTN3A1 | butyrophilin, subfamily 3, member A1 | 209770_at | 3.391 | 3.524 | 2.651 |
| GDF11 | growth differentiation factor 11 | 216860_s_at | 3.458 | 3.522 | 2.106 |
| IFIT5 | IFN-induced protein with tetratricopeptide repeats 5 | 203595_s_at | 3.503 | 3.516 | 3.442 |
| TNFRSF10A | tumor necrosis factor receptor superfamily, 10a | 231775_at | 3.246 | 3.497 | 2.008 |
| DCSTAMP | dendrocyte expressed seven transmembrane protein | 221266_s_at | 3.599 | 3.495 | 2.054 |
| SP100 | SP100 nuclear antigen | 202864_s_at | 3.262 | 3.484 | 2.802 |
| EIF2AK2 | eukaryotic translation initiation factor 2-alpha kinase 2 | 204211_x_at | 3.549 | 3.462 | 3.536 |
| PMAIP1 | phorbol-12-myristate-13-acetate-induced protein 1 | 204286_s_at | 2.957 | 3.412 | 2.028 |
| IRF9 | interferon regulatory factor 9 | 203882_at | 3.070 | 3.351 | 2.642 |
| TPCN1 | two pore segment channel 1 | 1557185_at | 3.201 | 3.276 | 2.032 |
| SMCHD1 | domain containing 1 | 212569_at | 3.165 | 3.259 | 2.168 |
| STS | steroid sulfatase (microsomal), isozyme S | 203767_s_at | 3.212 | 3.244 | 2.074 |
| LAP3 | leucine aminopeptidase 3 | 217933_s_at | 3.126 | 3.232 | 2.774 |
| TRIM22 | tripartite motif containing 22 | 213293_s_at | 3.111 | 3.198 | 2.878 |
| TNFAIP2 | tumor necrosis factor, alpha-induced protein 2 | 202510_s_at | 2.869 | 3.192 | 2.490 |
| EREG | epiregulin | 205767_at | 3.390 | 3.181 | 2.385 |
| ADAMTS20 | ADAM metallopeptidase with thrombospondin motif, 20 | 220717_at | 3.548 | 3.171 | 2.152 |
| TLR10 | toll-like receptor 10 | 223750_s_at | 3.019 | 3.144 | 2.264 |
| UBA7 | ubiquitin-like modifier activating enzyme 7 | 203281_s_at | 2.990 | 3.133 | 2.152 |
| DKFZp434J0226 | uncharacterized LOC93429 | 232832_at | 3.191 | 3.126 | 2.004 |
| PLSCR2 | phospholipid scramblase 2 | 207374_at | 2.975 | 3.124 | 2.663 |
| PLSCR1 | phospholipid scramblase 1 | 202430_s_at | 3.226 | 3.077 | 3.197 |
| MT1G | metallothionein 1G | 204745_x_at | 2.726 | 3.077 | 2.489 |
| GTPBP1 | GTP binding protein 1 | 205275_at | 2.988 | 3.062 | 2.041 |
| BTC | betacellulin | 241412_at | 2.556 | 3.028 | 2.358 |
| LOC100216545 | uncharacterized LOC100216545 | 238473_at | 2.969 | 3.020 | 2.338 |
| HLA-B | major histocompatibility complex, class I, B | 208729_x_at | 2.880 | 3.014 | 2.150 |
| FOXN2 | forkhead box N2 | 206708_at | 3.062 | 3.009 | 2.433 |
| PHF11 | PHD finger protein 11 | 221816_s_at | 2.783 | 2.994 | 2.244 |
| ACSL1 | acyl-CoA synthetase long-chain family member 1 | 207275_s_at | 2.779 | 2.974 | 2.109 |
| MT1F | metallothionein 1F | 217165_x_at | 2.514 | 2.954 | 2.322 |
| LAIR1 | leukocyte-associated immunoglobulin-like receptor 1 | 208071_s_at | 3.005 | 2.953 | 2.165 |
| MT1X | metallothionein 1X | 208581_x_at | 2.570 | 2.892 | 2.484 |
| GBP3 | guanylate binding protein 3 | 223434_at | 2.703 | 2.844 | 2.096 |
| LAMB4 | laminin, beta 4 | 234334_s_at | 2.823 | 2.784 | 2.147 |
| MT2A | metallothionein 2A | 212185_x_at | 2.515 | 2.776 | 2.238 |
| NHSL1 | NHS-like 1 | 234324_at | 3.217 | 2.751 | 2.423 |
| HFE | hemochromatosis | 211331_x_at | 2.826 | 2.744 | 2.175 |
| APBA2 | amyloid beta (A4) precursor protein-binding | 209871_s_at | 2.907 | 2.734 | 2.431 |
| RAB4B | RAB4B, member RAS oncogene family | 237257_at | 2.780 | 2.729 | 2.181 |
| UNC13D | unc-13 homolog D (C. elegans) | 226678_at | 2.856 | 2.714 | 2.092 |
| CSF1 | colony stimulating factor 1 (macrophage) | 209716_at | 2.566 | 2.705 | 2.096 |
| RABGAP1L | RAB GTPase activating protein 1-like | 215342_s_at | 2.393 | 2.681 | 2.013 |
| ZCCHC2 | zinc finger, CCHC domain containing 2 | 219062_s_at | 2.517 | 2.664 | 2.138 |
| ADAR | adenosine deaminase, RNA-specific | 201786_s_at | 2.565 | 2.659 | 2.262 |
| LRRC43 | leucine rich repeat containing 43 | 1553728_at | 2.690 | 2.643 | 2.147 |
| CXCL9 | chemokine (C-X-C motif) ligand 9 | 203915_at | 2.745 | 2.607 | 4.288 |
| NCOA7 | nuclear receptor coactivator 7 | 225344_at | 2.504 | 2.598 | 2.069 |
| CFHR2 | complement factor H-related 2 | 206910_x_at | 2.573 | 2.591 | 2.184 |
| APOH | apolipoprotein H (beta-2-glycoprotein I) | 231359_at | 2.586 | 2.551 | 2.076 |
| BTN3A2 | butyrophilin, subfamily 3, member A2 | 209846_s_at | 2.456 | 2.526 | 2.375 |
| GNB4 | guanine nucleotide binding protein (G protein), beta polypeptide 4 | 225710_at | 2.169 | 2.410 | 2.155 |
| ARHGEF40 | Rho guanine nucleotide exchange factor (GEF) 40 | 227855_at | 2.638 | 2.391 | 2.057 |
| SHISA5 | shisa homolog 5 (Xenopus laevis) | 222986_s_at | 2.289 | 2.352 | 2.492 |
| CTSS | cathepsin S | 202901_x_at | 2.254 | 2.337 | 2.004 |
| UNC93B1 | unc-93 homolog B1 (C. elegans) | 220998_s_at | 2.155 | 2.289 | 2.046 |
| REPS2 | RALBP1 associated Eps domain containing 2 | 227425_at | -2.297 | -2.406 | -2.096 |
| AK4 | adenylate kinase 4 | 230630_at | -2.340 | -2.428 | -3.953 |
| ITGAE | integrin, alpha E (antigen CD103, human mucosal lymphocyte antigen 1; alpha polypeptide) | 205055_at | -2.694 | -2.707 | -2.520 |
| LRPAP1 | low density lipoprotein receptor-related protein associated protein 1 | 235505_s_at | -2.506 | -2.803 | -2.311 |
| LOC100507303 | uncharacterized LOC100507303 | 228049_x_at | -2.680 | -2.921 | -2.038 |
| ENAH | enabled homolog (Drosophila) | 228310_at | -2.683 | -3.006 | -2.155 |
| C17orf89 | chromosome 17 open reading frame 89 | 225967_s_at | -2.765 | -3.080 | -2.158 |
| EPPIN | epididymal peptidase inhibitor | 206319_s_at | -3.344 | -3.210 | -2.147 |
| RPL37 | ribosomal protein L37 | 224763_at | -3.115 | -3.444 | -2.021 |
| DANCR | differentiation antagonizing non-protein coding RNA | 224870_at | -3.483 | -3.486 | -3.331 |
| CXCL14 | chemokine (C-X-C motif) ligand 14 | 222484_s_at | -2.629 | -3.518 | -2.589 |
| C7orf41 | chromosome 7 open reading frame 41 | 226018_at | -3.598 | -3.884 | -2.121 |
| MAP6 | microtubule-associated protein 6 | 228943_at | -4.579 | -4.049 | -2.511 |
| TTC3 | tetratricopeptide repeat domain 3 | 208663_s_at | -3.727 | -4.295 | -2.483 |
| DYNC2H1 | dynein, cytoplasmic 2, heavy chain 1 | 219469_at | -4.488 | -4.344 | -2.370 |
| WDR96 | WD repeat domain 96 | 231084_at | -6.128 | -4.849 | -2.034 |
| FMO5 | flavin containing monooxygenase 5 | 205776_at | -4.897 | -4.935 | -2.043 |
| SSR1 | signal sequence receptor, alpha | 225435_at | -4.500 | -5.001 | -2.042 |
| ZMAT3 | zinc finger, matrin-type 3 | 225725_at | -5.064 | -5.212 | -2.164 |
| CLN8 | ceroid-lipofuscinosis, neuronal 8 (epilepsy, progressive with mental retardation) | 229958_at | -5.249 | -5.421 | -2.753 |
| HELLS | helicase, lymphoid-specific | 227350_at | -5.259 | -5.828 | -4.433 |
| CD36 | CD36 molecule (thrombospondin receptor) | 228766_at | -5.736 | -6.030 | -2.469 |
| ZNF704 | zinc finger protein 704 | 235079_at | -5.438 | -6.132 | -4.822 |
| M1 | uncharacterized LOC100507027 | 231051_at | -6.641 | -6.216 | -2.260 |
| CMTM4 | CKLF-like MARVEL transmembrane domain containing 4 | 225009_at | -6.448 | -6.965 | -2.556 |
| ACO1 | aconitase 1, soluble | 207071_s_at | -6.544 | -7.267 | -3.448 |
