# Supplementary material for: Early Host Responses of Seasonal and Pandemic Influenza A Viruses in Primary Well-Differentiated Human Lung Epithelial Cells
Source: PLoS One. 2013 Nov 14;8(11):e78912. doi: 10.1371/journal.pone.0078912 (PMC3828299; doi:10.1371/journal.pone.0078912)
Supplement: Table S6 — Differentially expressed genes unique to BN/59 infected wd-NHBE cells at 36 hpi. (DOCX) [file pone.0078912.s009.docx]

**Table S6. Differentially expressed genes* unique to BN/59 infected wd-NHBE cells at 36 hpi**

| **Symbol** | **Entrez Gene Name** | **Affymetrix Probe** | **Fold Change** |
| --- | --- | --- | --- |
| POLDIP3 | polymerase (DNA-directed), delta interacting protein 3 | 210583_at | 3.60E-05 |
| POLRMT | polymerase (RNA) mitochondrial (DNA directed) | 203783_x_at | 1.11E-06 |
| WWOX | WW domain containing oxidoreductase | 223868_s_at | 2.89E-08 |

*DEGs determined by analysis conducted using Ingenuity core analysis (p<0.05, 2-fold change cut-off)
