# Supplementary material for: Early Host Responses of Seasonal and Pandemic Influenza A Viruses in Primary Well-Differentiated Human Lung Epithelial Cells
Source: PLoS One. 2013 Nov 14;8(11):e78912. doi: 10.1371/journal.pone.0078912 (PMC3828299; doi:10.1371/journal.pone.0078912)
Supplement: Table S7 — Top 25 significantly differentially expressed genes unique to KY/180 infected wd-NHBE cells at 36 hpi. (DOCX) [file pone.0078912.s010.docx]

**Table S7. Top 25 significantly differentially expressed genes* unique to KY/180 infected wd-NHBE cells at 36 hpi**

| **Symbol** | **Entrez Gene Name** | **Affymetrix Probe** | **Fold Change** |
| --- | --- | --- | --- |
| TRIB3 | tribbles homolog 3 (Drosophila) | 218145_at | 2.510 |
| AREG/AREGB | amphiregulin | 205239_at | 2.410 |
| CD53 | CD53 molecule | 203416_at | 2.324 |
| KRT20 | keratin 20 | 213953_at | 2.258 |
| mir-21 | microRNA 21 | 224917_at | 2.200 |
| COL4A6 | collagen, type IV, alpha 6 | 213992_at | 2.191 |
| LMO7 | LIM domain 7 | 242722_at | 2.175 |
| CEACAM3 | carcinoembryonic antigen-related cell adhesion molecule 3 | 217209_at | 2.156 |
| MFI2 | antigen p97 (melanoma associated) | 1556538_at | 2.151 |
| DRAXIN | dorsal inhibitory axon guidance protein | 1556826_s_at | 2.129 |
| PFKFB4 | 6-phosphofructo-2-kinase/fructose-2,6-biphosphatase 4 | 206246_at | 2.129 |
| DNAJB9 | DnaJ (Hsp40) homolog, subfamily B, member 9 | 202843_at | 2.106 |
| ART5 | ADP-ribosyltransferase 5 | 1552524_at | 2.101 |
| BDKRB1 | bradykinin receptor B1 | 207510_at | 2.098 |
| SYT15 | synaptotagmin XV | 1560879_a_at | 2.085 |
| COMMD5 | COMM domain containing 5 | 232640_at | 2.074 |
| GCOM1 | GRINL1A complex locus 1 | 228568_at | 2.071 |
| CASP5 | caspase 5, apoptosis-related cysteine peptidase | 207500_at | 2.056 |
| FOXE1 | forkhead box E1 (thyroid transcription factor 2) | 206912_at | 2.052 |
| LINC00184 | long intergenic non-protein coding RNA 184 | 1559646_a_at | 2.048 |
| SPTSSB | serine palmitoyltransferase, small subunit B | 238702_at | 2.048 |
| CALR | calreticulin | 214315_x_at | 2.043 |
| S100Z | S100 calcium binding protein Z | 1554876_a_at | 2.042 |
| SESN2 | sestrin 2 | 223195_s_at | 2.031 |
| SERPINB13 | serpin peptidase inhibitor, clade B (ovalbumin), member 13 | 211362_s_at | 2.030 |

*DEGs determined by analysis conducted using Ingenuity core analysis (p<0.05, 2-fold change cut-off)
