# Supplementary material for: Early Host Responses of Seasonal and Pandemic Influenza A Viruses in Primary Well-Differentiated Human Lung Epithelial Cells
Source: PLoS One. 2013 Nov 14;8(11):e78912. doi: 10.1371/journal.pone.0078912 (PMC3828299; doi:10.1371/journal.pone.0078912)
Supplement: Table S8 — Top 25 significantly differentially expressed genes unique to KY/136 infected wd-NHBE cells at 36 hpi. (DOCX) [file pone.0078912.s011.docx]

**Table S8. Top 25 significantly differentially expressed genes* unique to KY/136 infected wd-NHBE cells at 36 hpi**

| **Symbol** | **Entrez Gene Name** | **Affymetrix Probe** | **Fold Change** |
| --- | --- | --- | --- |
| MMP9 | matrix metallopeptidase 9 (gelatinase, 92kDa type IV collagenase) | 203936_s_at | 3.238 |
| GRIN3A | glutamate receptor, ionotropic, N-methyl-D-aspartate 3A | 233220_at | 3.048 |
| IFNA16 | interferon, alpha 16 | 208448_x_at | 2.957 |
| RGS1 | regulator of G-protein signaling 1 | 216834_at | 2.797 |
| OSR2 | odd-skipped related 2 (Drosophila) | 213568_at | 2.756 |
| COCH | coagulation factor C homolog, cochlin (Limulus polyphemus) | 205229_s_at | 2.693 |
| CCNA1 | cyclin A1 | 205899_at | 2.675 |
| TMPRSS3 | transmembrane protease, serine 3 | 220177_s_at | 2.601 |
| KCNS3 | potassium voltage-gated channel, subfamily S, member 3 | 205968_at | 2.556 |
| E2F5 | E2F transcription factor 5, p130-binding | 221586_s_at | 2.519 |
| RNF13 | ring finger protein 13 | 201780_s_at | 2.517 |
| LOC728743 | zinc finger protein pseudogene | 225909_at | 2.453 |
| FLJ32255 | uncharacterized LOC643977 | 235292_at | 2.439 |
| FOS | FBJ murine osteosarcoma viral oncogene homolog | 209189_at | 2.406 |
| IL12A | interleukin 12A (NK stimulatory and CTL maturation factor 1, p35) | 207160_at | 2.354 |
| RIPK1 | receptor (TNFRSF)-interacting serine-threonine kinase 1 | 209941_at | 2.317 |
| FBXO7 | F-box protein 7 | 201178_at | 2.311 |
| MB21D1 | Mab-21 domain containing 1 | 1559051_s_at | 2.276 |
| FAM43A | family with sequence similarity 43, member A | 227410_at | 2.275 |
| LOC100289019 | uncharacterized LOC100289019 | 1560089_at | 2.271 |
| OSMR | oncostatin M receptor | 1554008_at | 2.271 |
| ECM1 | extracellular matrix protein 1 | 209365_s_at | 2.258 |
| HCAR3 | hydroxycarboxylic acid receptor 3 | 205220_at | 2.252 |
| FYTTD1 | forty-two-three domain containing 1 | 224642_at | 2.251 |
| B3GNT7 | UDP-GlcNAc:betaGal beta-1,3-N-acetylglucosaminyltransferase 7 | 1555963_x_at | 2.248 |

NK (Natural Killer Cells); CTL (Cytotoxic T-lymphocyte)

*DEGs determined by analysis conducted using Ingenuity core analysis (p<0.05, 2-fold change cut-off)
