# Supplementary material for: Early Host Responses of Seasonal and Pandemic Influenza A Viruses in Primary Well-Differentiated Human Lung Epithelial Cells
Source: PLoS One. 2013 Nov 14;8(11):e78912. doi: 10.1371/journal.pone.0078912 (PMC3828299; doi:10.1371/journal.pone.0078912)
Supplement: Table S9 — Apoptosis genes differentially expressed at 36 hpi. (DOCX) [file pone.0078912.s012.docx]

**Table S9. Apoptosis genes differentially expressed at 36 hpi**

| **Symbol** | **Entrez Gene Name** | **Affymetrix Probe** | **Fold Change KY/180** | **Fold Change KY/136** | **Fold Change BN/59** |
| --- | --- | --- | --- | --- | --- |
| BAK1 | BCL2-antagonist/killer 1 | 203728_at | 3.80 | 4.37 | 1.89 |
| BCL2A1 | BCL2-related protein A1 | 205681_at | 11.29 | 12.05 | 2.57 |
| BIRC3 | baculoviral IAP repeat containing 3 | 210538_s_at | 5.42 | 6.07 | 3.12 |
| CAPN3 | calpain 3, (p94) | 210944_s_at | 2.06 | 2.20 | 1.22 |
| CASP7 | apoptosis related cysteine peptidase | 207181_s_at | 2.10 | 2.25 | 1.76 |
| CASP8 | apoptosis related Cysteine peptidase | 207686_s_at | 2.45 | 2.83 | 1.86 |
| CASP10 | apoptosis related cysteine peptidase | 205467_at | 2.9 | 3.50 | 1.69 |
| CDK1 | cyclin-dependent kinase 1 | 203213_at | -2.67 | -3.00 | -1.61 |
| CYCS | cytochrome c, somatic | 244546_at | -2.38 | -2.67 | -1.53 |
| DFFA | DNA fragmentation factor | 226116_at | -2.22 | -2.71 | -1.32 |
| ENDOG | endonuclease G | 204824_at | -2.52 | -2.43 | -1.37 |
| FAS | Fas, TNF receptor superfamily, member 6 | 216252_x_at | 2.28 | 2.65 | 1.70 |
| HTRA2 | HtrA serine peptidase | 2203089_s_at | -2.04 | -2.07 | -1.49 |
| IKBKE | inhibitor of kappa light polypeptide gene  enhancer in B-cells,  kinase epsilon | 204549_at | 2.12 | 2.40 | 1.62 |
| MCL1 | myeloid cell leukemia  sequence 1 (BCL2-  related) | 200798_x_at | 2.68 | 2.83 | 1.79 |
